# Cell surface flip-flop of phosphatidylserine is critical for PIEZO1-mediated myotube formation

**DOI:** 10.1038/s41467-018-04436-w

**Published:** 2018-05-24

**Authors:** Masaki Tsuchiya, Yuji Hara, Masaki Okuda, Karin Itoh, Ryotaro Nishioka, Akifumi Shiomi, Kohjiro Nagao, Masayuki Mori, Yasuo Mori, Junichi Ikenouchi, Ryo Suzuki, Motomu Tanaka, Tomohiko Ohwada, Junken Aoki, Motoi Kanagawa, Tatsushi Toda, Yosuke Nagata, Ryoichi Matsuda, Yasunori Takayama, Makoto Tominaga, Masato Umeda

**Affiliations:** 1 Department of Synthetic Chemistry and Biological Chemistry, Graduate School of Engineering, Kyoto University, Kyoto, 615-8510 Japan; 2 AMED-PRIME, Japan Agency for Medical Research and Development, Tokyo, 100-0004 Japan; 3 Department of Biology, Faculty of Sciences, Kyushu University, Fukuoka, 819-0395 Japan; 4 Institute for Integrated Cell-Material Sciences (WPI iCeMS), Kyoto University, Kyoto, 606-8501 Japan; 5 Physical Chemistry of Biosystems, Institute of Physical Chemistry, University of Heidelberg, Heidelberg, 69120 Germany; 6 Laboratory of Organic and Medicinal Chemistry, Graduate School of Pharmaceutical Sciences, The University of Tokyo, Tokyo, 115-0033 Japan; 7 Department of Molecular and Cellular Biochemistry, Graduate School of Pharmaceutical Sciences, Tohoku University, Miyagi, 980-8578 Japan; 8 Division of Neurology/Molecular Brain Science, Kobe University Graduate School of Medicine, Hyogo, 650-0017 Japan; 9 Department of Neurology, Division of Neuroscience, Graduate School of Medicine, The University of Tokyo, Tokyo, 113-8655 Japan; 10 Department of Life Science, Faculty of Science, Okayama University of Science, Okayama, 700-0005 Japan; 11 Department of Life Sciences, Graduate School of Arts and Sciences, The University of Tokyo, Tokyo, 153-8902 Japan; 12 Division of Cell Signaling, Okazaki Institute for Integrative Bioscience, Aichi, 444-8787 Japan; 13 Present Address: Center for Integrative Medicine and Physics, Institute for Advanced Study, Kyoto University, Kyoto, 606-8501 Japan

## Abstract

Myotube formation by fusion of myoblasts and subsequent elongation of the syncytia is essential for skeletal muscle formation. However, molecules that regulate myotube formation remain elusive. Here we identify PIEZO1, a mechanosensitive Ca^2+^ channel, as a key regulator of myotube formation. During myotube formation, phosphatidylserine, a phospholipid that resides in the inner leaflet of the plasma membrane, is transiently exposed to cell surface and promotes myoblast fusion. We show that cell surface phosphatidylserine inhibits PIEZO1 and that the inward translocation of phosphatidylserine, which is driven by the phospholipid flippase complex of ATP11A and CDC50A, is required for PIEZO1 activation. PIEZO1-mediated Ca^2+^ influx promotes RhoA/ROCK-mediated actomyosin assemblies at the lateral cortex of myotubes, thus preventing uncontrolled fusion of myotubes and leading to polarized elongation during myotube formation. These results suggest that cell surface flip-flop of phosphatidylserine acts as a molecular switch for PIEZO1 activation that governs proper morphogenesis during myotube formation.

## Abstract (web summary)

Myotube formation by fusion of myoblasts is essential for skeletal muscle formation, but which molecules regulate this process remains elusive. Here authors identify the mechanosensitive PIEZO1 channel as a key element, whose activity is regulated by phosphatidylserine during myotube formation.

## Introduction

Transbilayer relocation of phospholipids at the plasma membrane is critical for various cellular processes such as cell division, signal transduction, and vesicular transport^1–4^. Phosphatidylserine (PS), a negatively charged phospholipid, normally resides in the inner leaflet of the plasma membrane^5^. Controlled cell surface exposure of PS acts as a potent promoter of blood coagulation, apoptotic cell engulfment, and myogenesis^6–9^. Mammalian skeletal muscles are formed by the fusion of mononucleated precursor cells (myoblasts) into unusually elongated multinucleated cells called myotubes, whose formation relies on orchestrated cell-to-cell fusion and elongation of multinucleated syncytia^10, 11^. During myotube formation, PS transiently translocates to the outer leaflet of the plasma membrane and recognition of cell surface-exposed PS by PS receptors induces contact-dependent signaling to promote fusion with neighboring myoblasts^9, 12–15^. However, it remains unclear how the transbilayer relocation of PS at the plasma membrane is controlled during myotube formation.

Several members of the type IV subfamily of P-type adenosine triphosphatases (P4-ATPases) that are complexed with an auxiliary CDC50 subunit act as a phospholipid flippase that translocates the cell surface-exposed PS to the inner leaflet of the plasma membrane^3, 4, 8, 16–18^. In mammals, at least 14 members of P4-ATPases, designated ATP8A1 through ATP11C, and three CDC50 family proteins (CDC50A, CDC50B, and CDC50C) have been identified^3, 4, 17, 18^. ATP8A1, ATP8A2, ATP8B1, ATP8B2, ATP8B4, ATP10A, ATP10D, ATP11A, and ATP11C are localized to the plasma membrane, whereas ATP9A, ATP9B, ATP10B, and ATP11B are distributed to intracellular membranes^3, 4, 8, 16–18^. Among the cell surface-localized P4-ATPases, ATP8A1, ATPA2, ATP8B1, ATP11A, and ATP11C have been shown to catalyze the inward translocation of PS at the plasma membrane^3, 4, 8, 16–18^. As first reported in yeast^4, 19^, complex association with CDC50 family proteins is required for transport of these P4-ATPases from endoplasmic reticulum to the plasma membrane, where they play a dominant role in maintaining the asymmetric distribution of PS in the bilayer leaflet^3, 4, 8, 16–18, 20^.

Although little is known about the physiological functions of mammalian P4-ATPases, deficiencies of at least three P4-ATPases, ATP8A2, ATP8B1, and ATP11C, can cause severe human disease^3, 17, 18, 21, 22^. Mutations identified in *ATP8A2*, which is highly expressed in the brain, testes, and retina, are associated with cerebellar ataxia, mental retardation and disequilibrium syndrome^17, 18^. Mutations in *ATP8B1* cause liver disorders such as progressive familial intrahepatic cholestasis type 1 (PFIC1) and benign recurrent intrahepatic cholestasis type 1 (BRIC1)^17^. A mutation in *ATP11C* is liked to a congenital hemolytic anemia^22^. In vivo studies in mouse models have also contributed to our understanding of the physiological functions of mammalian P4-ATPases: *Atp8a1*-deficient mice exhibit delayed hippocampus-dependent learning, *Atp8a2*-mutant mice display neurological abnormalities such as ataxia derived from axonal degeneration and loss of visual/auditory functions, and *Atp11c-*mutant mice show arrested B cell development^17, 21^. ATP11A is ubiquitously expressed in various tissues^16^ and deletion of *Atp11a* results in lethality during embryogenesis^23^. The function of P4-ATPases in skeletal muscle, however, remains to be elucidated.

Here we identify the phospholipid flippase complex of ATP11A and CDC50A as a critical regulator for activation of the mechanosensitive Ca^2+^ channel PIEZO1^24, 25^ during myotube formation. We show that the phospholipid flippase-mediated translocation of cell surface-exposed PS is a prerequisite for activation of PIEZO1 and that PIEZO1-mediated Ca^2+^ influx promotes RhoA/ROCK-dependent actomyosin assemblies^26^, thus leading to controlled cell fusion and the polarized elongation of multinucleated myotubes. The inhibitory effect of cell surface-exposed PS on PIEZO1 is strictly dependent on the headgroup structure of PS, and is controlled by manipulating the amount of PS present on the cell surface. Furthermore, the myoblast-specific disruption of *Atp11a* results in the formation of abnormal myofibres that fuse with each other during muscle regeneration after injury.

## Results

### ATP11A/CDC50A is required for myotube formation

**Fig. 1:**
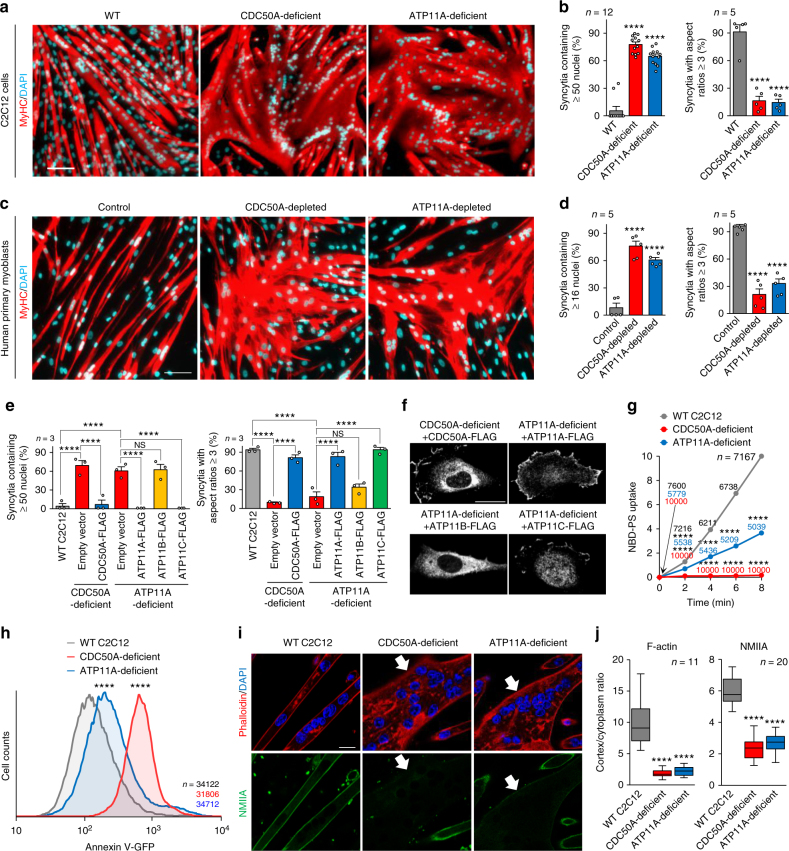
Defective myotube formation by CDC50A-deficient and ATP11A-deficient myoblasts. **a**–**d** Aberrant morphologies of PS flippase-deficient myotubes. **a** Syncytia formed by WT and CDC50A-deficient or ATP11A-deficient C2C12 myoblasts were visualized by immunofluorescent staining with anti-MyHC antibody (differentiated cells, red) and DAPI (nuclei, cyan). **b** Left: cell fusion evaluated as percentages of syncytia containing ≥50 nuclei in **a**. Right: polarized elongation evaluated as percentages of syncytia with aspect ratios ≥3 in **a**. **c** Syncytia formed by human primary myoblasts transfected with control, CDC50A or ATP11A siRNA were visualized by immunofluorescent staining with anti-MyHC antibody and DAPI. **d** Left: cell fusion evaluated as percentages of syncytia containing ≥16 nuclei in **c**. Right: polarized elongation evaluated as percentages of syncytia with aspect ratios ≥3 in **c**. **e**, **f** Rescue of morphologies in PS flippase-deficient myotubes by overexpression of PS flippase complex components. **e** Syncytia formed by WT, CDC50A-deficient or ATP11A-deficient C2C12 myoblasts expressing FLAG-tagged CDC50A, ATP11A, ATP11B or ATP11C were visualized by immunofluorescent staining with anti-MyHC antibody and DAPI in Supplementary Figure 3b. Left: cell fusion evaluated as percentages of syncytia containing ≥50 nuclei. Right: polarized elongation evaluated as percentages of syncytia with aspect ratios ≥3. **f** Localization of FLAG-tagged proteins expressed in CDC50A-deficient or ATP11A-deficient C2C12 myoblasts. **g**, **h** PS exposure on PS flippase-deficient myoblasts. **g** Flow cytometry analysis of inward translocation (flip) activity of fluorescence-labelled PS (NBD-PS) at the plasma membrane of WT, CDC50A-deficient and ATP11A-deficient C2C12 myoblasts. **h** Flow cytometry histogram of WT, CDC50A- and ATP11A-deficient C2C12 myoblasts labelled with annexin V-GFP. **i**, **j** Mislocalization of cortical actomyosin in PS flippase-deficient myotubes. **i** Localization of F-actin (phalloidin, red) and NMIIA (anti-NMIIA antibody, green) in WT, CDC50A-deficient and ATP11A-deficient C2C12 syncytia. Arrows indicate syncytia with diminished peripheral NMIIA accumulation. **j** Cortex vs. cytoplasm ratio of F-actin and NMIIA signals in **i**. ****P < 0.0001 (Student’s *t*-test). NS not significant, *n* sample number. Bar graphs represent mean ± S.E.M. Box and whiskers graph-line: median, box: upper and lower quartiles, whiskers: maxima and minima. Scale bars: 100 μm (**a**, **c**), 20 μm (**f**, **i**)

In this study, we detected the expression of seven genes encoding P4-ATPases and a single gene for a CDC50 family protein, CDC50A, in mouse C2C12 myoblasts^12, 27^ (Supplementary Figure 1a–c). Under myogenic differentiation conditions as indicated by the expression of muscle-type myosin heavy chain (MyHC), C2C12 myoblasts changed cell shape to a bipolar form and fused to generate elongated multinucleated myotubes, thereby providing a quantifiable in vitro model of myogenesis (Fig. 1a). CDC50A-deficient C2C12 cells produced by the CRISPR/Cas9 system^28^ (Supplementary Figure 1d) differentiated and fused with neighboring cells, but formed aberrantly enlarged syncytia with an undefined cell shape (Fig. 1a). Nuclei number and aspect ratios of the syncytia indicated excessive cell fusion and defects in polarized elongation in the CDC50A-deficient cells (Fig. 1b and Supplementary Figure 1e). Among the P4-ATPases expressed in C2C12 cells, ATP8B1, ATP8B2, ATP11A, ATP11B, and ATP11C were found to associate with CDC50A (Supplementary Figure 1f). We then established a series of P4-ATPase-deficient cell lines (Supplementary Figure 1d) and demonstrated that ATP11A-deficient cells formed enlarged syncytia with morphological features similar to those of the CDC50A-deficient cells (Fig. 1a, b and Supplementary Figure 1e). Live-cell imaging showed that neighboring C2C12 myoblasts fused with each other, and the resulting syncytia elongated longitudinally to form mature myotubes (Supplementary Figure 1g and Supplementary Movie 1). In contrast, ATP11A-deficient and CDC50A-deficient cells excessively fused with neighboring myoblasts, and the resulting syncytia spread without polarized elongation, forming an enlarged sheet-like morphology (Supplementary Figure 1g, Supplementary Movies 2 and 3). These observations were further confirmed using human primary myoblasts in which the phospholipid flippase complex of ATP11A and CDC50A was also robustly expressed (Supplementary Figure 2a). siRNA-mediated depletion of either CDC50A or ATP11A caused similar morphological abnormalities in syncytia without affecting differentiation (Fig. 1c, d and Supplementary Figure 2b, c). Retrovirus-mediated gene transfer was conducted to confirm the role of the phospohlipid flippase complex in myotube formation. Morphological abnormalities in the CDC50A-deficient or ATP11A-deficient C2C12 syncytia were rescued by re-expression of FLAG-tagged CDC50A or ATP11A, respectively (Fig. 1e, f and Supplementary Figure 3a–c). Importantly, ATP11C (a P4-ATPase localized to the plasma membrane like ATP11A) prevented the morphological abnormalities seen in the ATP11A-deficient C2C12 syncytia, but ATP11B residing in intracellular membranes did not (Fig. 1e, f and Supplementary Figure 3a–c).

We analyzed the inward translocation activity of PS at the plasma membrane using the fluorescence-labelled PS analogue NBD-PS^8, 20^. After incubation of cells for various amounts of time with NBD-PS, we removed the fluorescent lipid remaining in the outer leaflet by washing with lipid-free bovine serum albumin (BSA). Flow cytometric analysis of cells incorporating NBD-PS into the inner leaflet showed that PS translocation was significantly reduced in both CDC50A-deficient and ATP11A-deficient C2C12 cells (Fig. 1g). Flow cytometric analysis of PS-exposing cells labelled with the PS-binding protein annexin V^8^ (Fig. 1h), fluorescent spectroscopic analysis using the surface charge-sensitive probe F2N12S^29^ (Supplementary Figure 1h) and quantification of PS contents by thin-layer chromatography (Supplementary Figure 1i) demonstrated that PS was exposed on the cell surface in CDC50A-deficient and ATP11A-deficient cells, without changes in PS content. Although previous studies^9, 12–15^ have shown that PS is transiently exposed on the cell surface during myotube formation, the molecular mechanisms underlying PS exposure remain unclear. In relation to this event, caspases are transiently activated in the early phase of myotube formation^30, 31^. Although caspase-mediated degradation of ATP11A has been shown to be responsible for PS exposure during apoptosis^16^, it remains unclear whether ATP11A degradation is involved in PS exposure during myotube formation. Here we found that ATP11A expression was significantly reduced in the early phase of C2C12 cell differentiation and restored by the presence of a caspase inhibitor, suggesting that the caspase-mediated transient cleavage of ATP11A is responsible for PS exposure on the cell surface (Supplementary Figure 1j). Taken together, these results suggest that the clearance of cell surface-exposed PS by the phospholipid flippase complex of ATP11A and CDC50A (PS flippase) plays a crucial role in the myotube formation. During myotube formation, assemblies of F-actin and non-muscle myosin IIA (NMIIA) create actomyosin fibers underneath the plasma membrane, which prevents uncontrolled fusion of adjacent myotubes and generates a lateral compression force to support polarized elongation^10, 32^. Both F-actin and NMIIA were enriched at the lateral cortex of wild-type (WT) C2C12 myotubes, but neither significant accumulation of F-actin nor NMIIA to the cell periphery was observed in the sheet-like syncytium of the CDC50A-deficient and ATP11A-deficient cells (Fig. 1i). Analysis of actomyosin localization by quantifying cortex/cytoplasm ratios^33^ clearly demonstrated the suppressed assembly of cortical F-actin and NMIIA in the sheet-like syncytium, which accumulated in unfused mononuclear cells (Fig. 1i, j).

### PIEZO1 is required for myotube formation

**Fig. 2:**
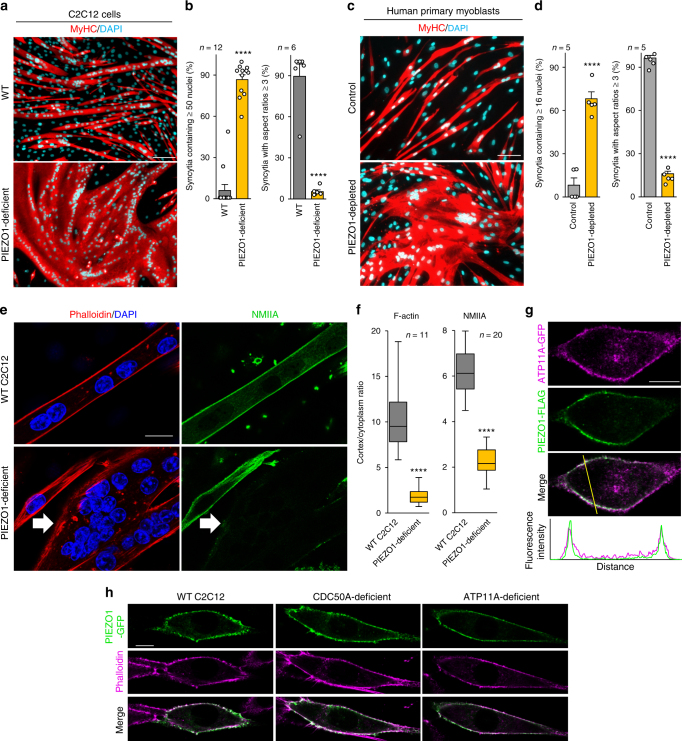
Defective myotube formation by PIEZO1-deficient myoblasts. **a**–**d** Aberrant morphologies of PIEZO1-deficient myotubes. **a** Syncytia formed by WT and PIEZO1-deficient C2C12 myoblasts were visualized by immunofluorescent staining with anti-MyHC antibody (differentiated cells, red) and DAPI (nuclei, cyan). **b** Left: cell fusion evaluated as percentages of syncytia containing ≥50 nuclei in **a**. Right: polarized elongation evaluated as percentages of syncytia with aspect ratios ≥3 in **a**. **c** Syncytia formed by human primary myoblasts transfected with control or PIEZO1 siRNA were visualized by immunofluorescent staining with anti-MyHC antibody (differentiated cells, red) and DAPI (nuclei, cyan). **d** Left: cell fusion evaluated as percentages of syncytia containing ≥16 nuclei in **c**. Right: polarized elongation evaluated as percentages of syncytia with aspect ratios ≥3 in **c**. **e**, **f** Mislocalization of cortical actomyosin in PIEZO1-deficient myotubes. **e** Localization of F-actin (phalloidin, red) and NMIIA (anti-NMIIA antibody, green) at the cell periphery of WT and PIEZO1-deficient C2C12 syncytia. Arrows indicate PIEZO1-deficient syncytia with diminished peripheral accumulation of NMIIA. **f** Cortex vs. cytoplasm ratio of F-actin and NMIIA signals in **e**. **g**, **h** Normal cell surface expression of PIEZO1 in PS flippase-deficient myoblasts. **g** Co-localization of GFP-tagged ATP11A (magenta) and FLAG-tagged PIEZO1 (green) in WT C2C12 myoblasts. Merged images and signal intensities are shown in the bottom panels. **h** Co-localization of GFP-tagged PIEZO1 (anti-GFP antibody, green) and F-actin (phalloidin, magenta) at the cell periphery of WT, CDC50A-deficient and ATP11A-deficient C2C12 myoblasts. Merged images are shown in the bottom panel. ****P < 0.0001 (Student’s *t*-test). *n* sample number. Bar graphs represent mean ± S.E.M. Box and whiskers graph-line: median, box: upper and lower quartiles, whiskers: maxima and minima. Scale bars: 100 μm (**a**, **c**), 20 μm (**e**), 10 μm (**g**, **h**)

Myotube formation and the accompanying cortical actomyosin assembly are dependent on the influx of Ca^2+^ across the plasma membrane^11^. Because mechanical tension is crucial for myoblast fusion events^34^, we examined the effect of the depletion of mechanosensitive Ca^2+^-permeable channels expressed in C2C12 myoblasts on myotube formation (Supplementary Figure 4a). Of the Ca^2+^ channels examined, siRNA-mediated depletion of PIEZO1^24, 25^, a mechanosensitive Ca^2+^ channel predominantly expressed during myotube formation, resulted in the formation of sheet-like syncytia (Supplementary Figure 4a, b), showing a morphological phenotype quite similar to that observed in the ATP11A-deficient and CDC50A-deficient cells (Fig. 1a). Thus, we established PIEZO1-deficient C2C12 cells using the CRISPR/Cas9 system^28^ (Supplementary Figure 4c), in which the mutations completely abolished the function of PIEZO1 as a Ca^2+^ channel (Supplementary Figure 4d–f). Quantitative analyses of syncytium morphology (Fig. 2a, b and Supplementary Figure 4g, h) and time-lapse observations (Supplementary Movie 4) confirmed excessive cell fusion and the cell-elongation defect in PIEZO1-deficient C2C12 cells. siRNA-mediated depletion of PIEZO1 in human primary myoblasts also caused morphological abnormalities in myotube formation similar to those observed in PS flippase-deficient cells (Fig. 2c, d and Supplementary Figure 2a–c). Moreover, there was no significant accumulation of cortical F-actin or NMIIA in the syncytium formed by the PIEZO1-deficient C2C12 cells (Fig. 2e, f). These results suggest that both PS flippase and PIEZO1 are involved in the cortical actomyosin assembly, as well as in myotube formation.

### PS flippase is required for PIEZO1 activation

**Fig. 3:**
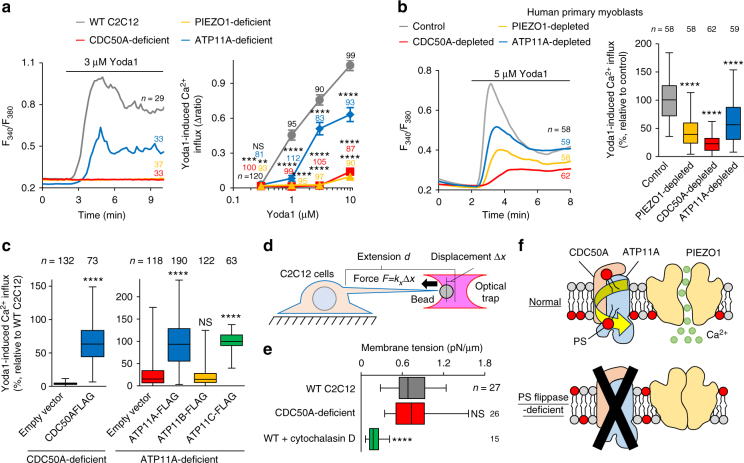
Impaired PIEZO1 activation in PS flippase-deficient myoblasts. **a**, **b** Suppression of agonist-induced PIEZO1 activation by PS flippase deficiency. **a** Left: Fura2 ratiometric measurements (F_340_/F_380_) of Yoda1-induced Ca^2+^ influx in WT, PIEZO1-deficient, CDC50A-deficient, and ATP11A-deficient C2C12 myoblasts. Right: quantification of Yoda1-induced Ca^2+^ influx as the maximal increment of F_340_/F_380_ (∆ ratio) in left. **b** Left: Fura2 ratiometric measurements of Yoda1-induced Ca^2+^ influx in human primary myoblasts transfected with control, PIEZO1, CDC50A or ATP11A siRNA. Right: quantification of Yoda1-induced Ca^2+^ influx in left. **c** Rescue of agonist-induced PIEZO1 activation in PS flippase-deficient myoblasts by overexpression of PS flippase complex components. Quantification of Yoda1-induced Ca^2+^ influx in CDC50A-deficient (left) or ATP11A-deficient (right) C2C12 myoblasts expressing FLAG-tagged CDC50A, ATP11A, ATP11B or ATP11C. **d**, **e** Normal plasma membrane tension in PS flippase-deficient myoblasts. **d** Schematic model of membrane tension measurement using an optical trap. **e** Quantification of membrane tension in WT, CDC50A-deficient and cytochalasin D-treated WT C2C12 cells. **f** Schematic model showing PS flippase-mediated inward translocation of cell surface-exposed PS as a prerequisite for PIEZO1 activation. **P < 0.01, ***P < 0.001, and ****P < 0.0001 (Student’s *t*-test). NS not significant, *n* sample number. Box and whiskers graph―line: median, box: upper and lower quartiles, whiskers: maxima and minima

To eliminate the possible effect of defective PS flippase expression on translocation of PIEZO1 to the cell surface, we examined the cellular localization of PIEZO1 in flippase-deficient C2C12 cells. Both ATP11A and PIEZO1 were predominantly localized on the plasma membrane (Fig. 2g), and deficiency of CDC50A or ATP11A had no significant effect on the cell surface localization of PIEZO1 (Fig. 2h). Next, PIEZO1-mediated Ca^2+^ influx was monitored by Fura2 ratiometric imaging using the PIEZO1 activator Yoda1, which mimics mechanical activation^35, 36^. Ca^2+^ influx in response to Yoda1 was completely abolished in the CDC50A-deficient C2C12 cells and significantly reduced in the ATP11A-deficient cells (Fig. 3a). The inhibitory effect on PIEZO1 activation was also observed in human primary myoblasts transfected with siRNA against CDC50A or ATP11A (Fig. 3b). PIEZO1-mediated Ca^2+^ influx in the CDC50A-deficient or ATP11A-deficient C2C12 cells was recovered by exogenous expression of CDC50A or one of the PS flippases (i.e., ATP11A or ATP11C^8^), respectively (Fig. 3c and Supplementary Figure 5a). PIEZO1 overexpression restored the Yoda1-elicited Ca^2+^ influx in the PIEZO1-deficient C2C12 cells, but not in the CDC50A-deficient cells (Supplementary Figure 5b).

Because lateral membrane tension is the physical stimulus that activates PIEZO1^37^, we next examined whether depletion of PS flippase might affect plasma membrane tension. Membrane tension was measured by pulling a thin tube of membrane from the cell surface with an adhesive polystyrene bead in an optical trap^38^ (Fig. 3d). No significant change was observed between WT and CDC50A-deficient C2C12 cells (Fig. 3e and Supplementary Figure 5c). In contrast, inhibition of actin polymerization by treatment with cytochalasin D significantly reduced membrane tension, as previously reported^38^ (Fig. 3e and Supplementary Figure 5c). These results raise the intriguing possibility that the diminished response of PIEZO1 in PS flippase-deficient cells is due to the change in the transbilayer distribution of PS at the plasma membrane (Fig. 3f).

### Cell surface flip-flop of PS regulates PIEZO1 activity

**Fig. 4:**
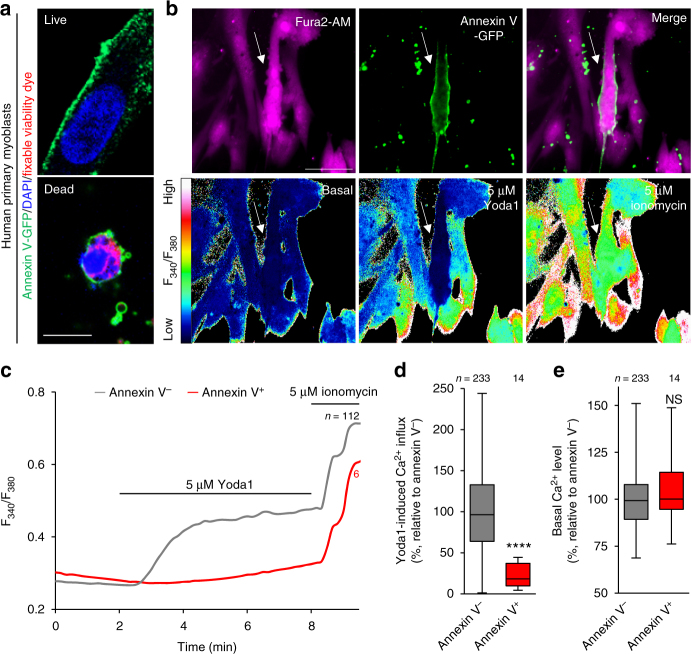
Impaired PIEZO1 activation in PS-exposing human primary myoblasts during myotube formation. **a** Detection of cell surface-exposed PS on differentiating human primary myoblasts by annexin V-GFP (PS, green), DAPI (nuclei, blue) and fixable viability dye (dead cells, red). **b** Fura2 imaging of Ca^2+^ influx in annexin V-GFP-labelled human primary myoblasts (arrow) upon addition of Yoda1 and ionomycin. Representative traces (**c**), quantification of Yoda1-induced Ca^2+^ influx (**d**) and basal Ca^2+^ level (**e**) in **b**. ****P < 0.0001 (Student’s *t*-test). NS not significant, *n* sample number. Box and whiskers graph―line: median, box: upper and lower quartiles, whiskers: maxima and minima. Scale bars: 10 μm (**a**), 20 μm (**b**)

**Fig. 5:**
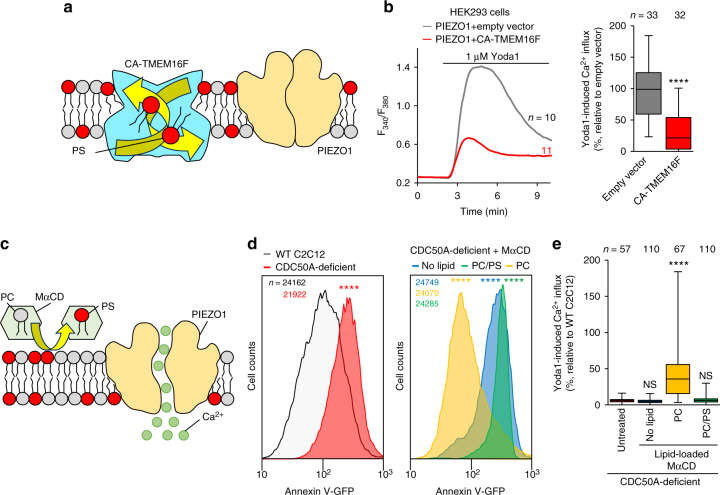
Suppression of PIEZO1 activation by cell surface-exposed PS. **a**, **b** Suppression of agonist-induced PIEZO1 activation by overexpression of phospholipid scramblases. **a** Schematic illustration showing inhibition of PIEZO1 activation by CA-TMEM16-mediated bidirectional translocation of PS in the plasma membrane. **b** Representative traces (left) and quantification (right) of Yoda1-induced Ca^2+^ influx in HEK293 cells co-expressing PIEZO1 and CA-TMEM16F. **c**–**e** Rescue of agonist-induced PIEZO1 activation in PS flippase-deficient myoblasts by MαCD-catalyzed replacement of cell surface-exposed PS with exogenous PC. **c** Schematic illustration showing restoration of PIEZO1 activation by phospholipid exchange between PC-loaded MαCD and PS-exposing myoblasts. **d**, **e** Flow cytometry histograms of annexin V-GFP labelling (**d**) and quantification of Yoda1-induced Ca^2+^ influx (**e**) of CDC50A-deficient C2C12 myoblasts pre-incubated with no lipid-, PC/PS mixture (3:1)- or PC-loaded MαCD. ****P < 0.0001 (Student’s *t*-test). NS not significant, *n* sample number. Box and whiskers graph-line: median, box: upper and lower quartiles, whiskers: maxima and minima

Next, we investigated the possible inhibitory effect of cell-surface-exposed PS on PIEZO1 activation in the course of myotube formation. Consistent with the results obtained from mouse differentiating myoblasts^9, 12–15^, cell surface exposure of PS was clearly observed on viable human primary myoblasts, accounting for about 20% of differentiating myoblasts (Fig. 4a). In PS-exposing myoblasts, PIEZO1-mediated Ca^2+^ influx was strikingly impaired (Fig. 4b–d), whereas both the basal Ca^2+^ concentration and ionomycin-induced Ca^2+^ influx were normal (Fig. 4c, e). To assess whether inward translocation of PS at the plasma membrane is a prerequisite for PIEZO1 activation, the transbilayer distribution of PS was manipulated by co-expression of PIEZO1 with a constitutively active form of TMEM16F (CA-TMEM16F), a phospholipid scramblase that induces cell-surface exposure of PS by bidirectional transport of phospholipids between the bilayer leaflets^7, 39^ (Fig. 5a). Ca^2+^ influx via PIEZO1 was significantly suppressed by the expression of CA-TMEM16F, compared to that of empty vector-transfected HEK293 cells (Fig. 5b). In addition, the inhibitory effect of cell-surface PS was further confirmed by manipulating the cell-surface contents of PS using the methyl-α-cyclodextrin (MαCD)-catalyzed phospholipid exchange method^40^ (Fig. 5c, d). PIEZO1-mediated Ca^2+^ influx in CDC50-deficient C2C12 cells was significantly recovered when cell surface-exposed PS was exchanged with exogenous phosphatidylcholine (PC), but not with a mixture containing PS (PC/PS) (Fig. 5e). These results indicate that PS flippase-mediated inward translocation of cell surface PS, which was exposed during myotube formation^9, 12–15^, is essential for PIEZO1 activation.

**Fig. 6:**
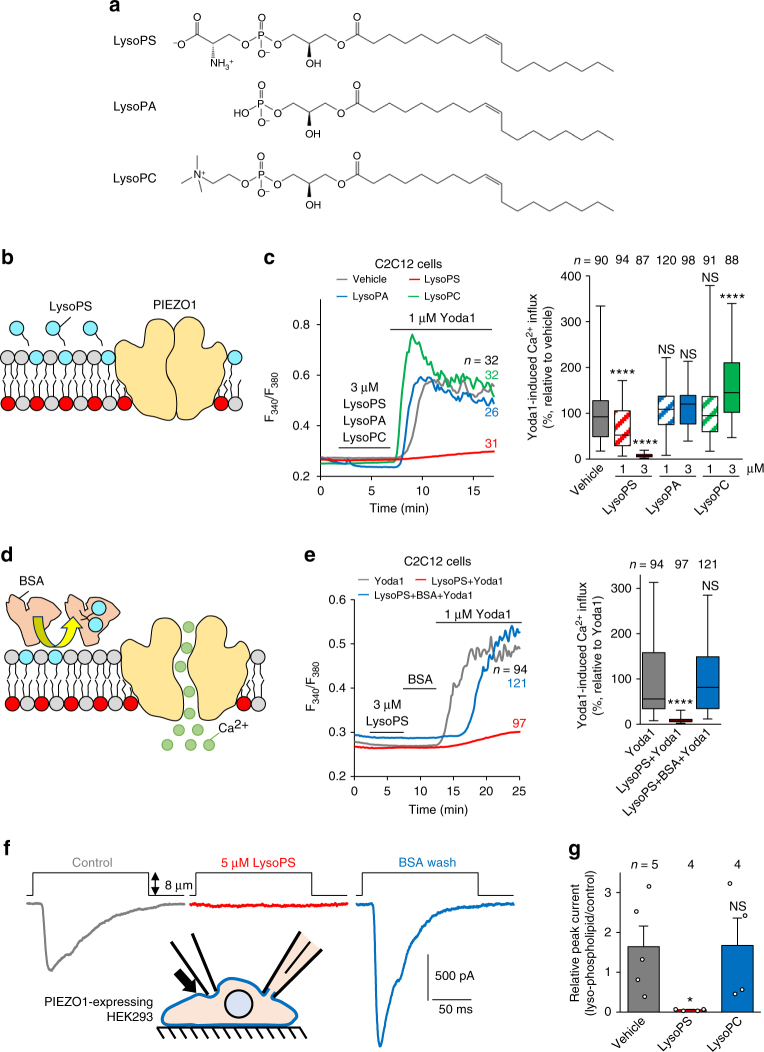
Suppression of PIEZO1 activation by cell surface-inserted LysoPS. **a** Chemical structures of LysoPS, LysoPA and LysoPC. **b**–**d** Suppression of agonist-induced PIEZO1 activation by cell-surface LysoPS. **b** Schematic model showing suppression of PIEZO1 activation by insertion of LysoPS to the cell surface. **c** Representative traces (left) and quantification (right) of Yoda1-induced Ca^2+^ influx in WT C2C12 myoblasts treated with vehicle, LysoPS, LysoPA or LysoPC. **d** Schematic model showing restoration of PIEZO1 activation by removal of cell surface-inserted LysoPS with lipid-free BSA. **e** Representative traces (left) and quantification (right) of Yoda1-induced Ca^2+^ influx in WT C2C12 myoblasts treated with LysoPS and washed with lipid-free BSA. **f**, **g** Impairment of mechanical stimulation-induced PIEZO1 activation by cell-surface LysoPS. **f** Representative traces of the mechanically-activated current evoked by indentation (8 μm) using a glass probe in PIEZO1-expressing HEK293 cells before (grey, control) and during administration of 5 μM LysoPS (red), followed by removal of LysoPS with lipid-free BSA (blue). **g** Relative peak currents induced by mechanical stimulation before (control) and during administration of vehicle (grey), 5 μM LysoPS (red), and 5 μM LysoPC (blue) in **f**. *P < 0.05 and ****P < 0.0001 (Student’s *t*-test). NS not significant, *n* sample number. Bar graphs represent mean ± S.E.M. Box and whiskers graph―line: median, box: upper and lower quartiles, whiskers: maxima and minima

The direct effect of cell surface-exposed PS was further examined by treatment with a deacylated form of PS, lyso-phosphatidylserine (LysoPS), which readily inserts into the outer leaflet of the plasma membrane^41^ (Fig. 6a, b). Ca^2+^ influx via PIEZO1 was dose-dependently suppressed by the incorporation of LysoPS into the plasma membrane of C2C12 cells, while the incorporation of a zwitterionic phospholipid, lyso-phosphatidylcholine (LysoPC), or an anionic phospholipid, lyso-phosphatidic acid (LysoPA), showed no significant inhibitory effect on Ca^2+^ influx (Fig. 6a, c). Furthermore, slight modification of the serine moiety of LysoPS abolished its ability to inhibit PIEZO1 activity, suggesting that the stereospecific inhibition of PIEZO1 is mediated by the serine headgroup of PS (Supplementary Figure 5d). Ca^2+^ influx via PIEZO1 was restored when LysoPS on the cell surface was removed by washing with lipid-free BSA^8^ (Fig. 6d, e). To confirm the inhibitory effect of LysoPS on membrane tension-induced PIEZO1 activation^37^, we conducted electrophysiological measurements of the PIEZO1 current by applying mechanical force to the cell surface using an electrically-driven glass probe^24^. In PIEZO1-expressing HEK293 cells, indentation of the cell surface by 8 μm evoked a transient inward current, consistent with that observed in a previous study^24^ (Fig. 6f). The addition of LysoPS significantly blunted the mechanically gated currents, while removal of cell surface-inserted LysoPS with BSA completely restored the responsiveness of PIEZO1 to mechanical stimuli (Fig. 6f). No significant change in the current was observed with the addition of LysoPC (Fig. 6g). In addition, application of negative pressure (−20 mmHg) to the cell surface evoked a transient inward current in PIEZO1-expressing cells, and the addition of LysoPS in the recording pipettes significantly blunted the PIEZO1 currents (Supplementary Figure 5e). These data suggest that the phospholipids with phosphoserine headgroups present on the outer leaflet of the plasma membrane are responsible for inhibition of PIEZO1 activation.

### PS flippase and PIEZO1 regulate cortical actomyosin assembly

**Fig. 7:**
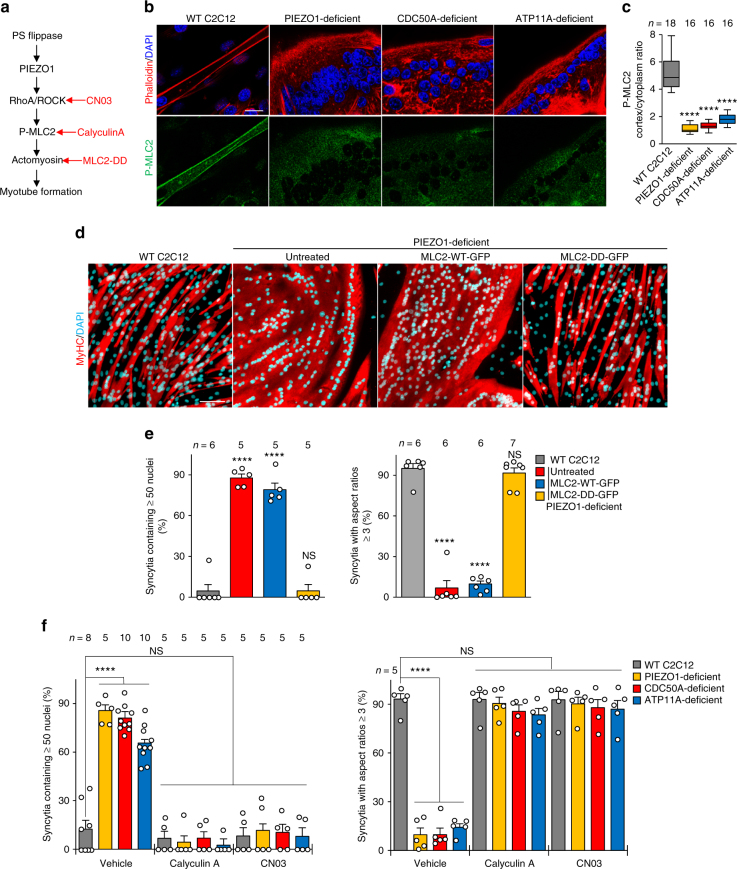
RhoA/ROCK-mediated actomyosin formation via the PS flippase/PIEZO1 pathway required for myotube morphology. **a** Schematic diagram of the PS flippase/PIEZO1 axis (black) and activators for the RhoA/ROCK/actomyosin pathway (red). **b**, **c** Suppressed cortical accumulation of phosphorylated MLC2 (P-MLC2) in PS flippase-deficient and PIEZO1-deficient myotubes. **b** Localization of P-MLC2 (anti-P-MLC2 antibody, green), F-actin (phalloidin, red), and nuclei (DAPI, cyan) in WT, PIEZO1-deficient, CDC50A-deficient, and ATP11A-deficient C2C12 syncytia. Intense co-localization of P-MLC2 and F-actin was observed on the cell periphery of WT myotubes. **c** Cortex vs. cytoplasm ratio of P-MLC2 signals in **b**. **d**, **e** Rescue of myotube formation by stable expression of a phospho-mimetic form of MLC2 in PIEZO1-deficient C2C12 syncytia. **d** Syncytia formed by WT and PIEZO1-deficient C2C12 myoblasts stably expressing WT MLC2 (MLC2-WT-GFP) or phospho-mimetic MLC2 (MLC2-DD-GFP) were visualized by immunofluorescent staining with anti-MyHC antibody (differentiated cells, red) and DAPI (nuclei, cyan). **e** Left: cell fusion evaluated as percentages of syncytia containing ≥50 nuclei in **d**. Right: polarized elongation evaluated as percentages of syncytia with aspect ratios ≥3 in **d**. **f** Rescue of myotube formation by activation of the RhoA/ROCK/actomyosin pathway in PS flippase- or PIEZO1-deficient C2C12 syncytia. Syncytia formed by WT, PIEZO1-deficient, CDC50A-deficient, and ATP11A-deficient C2C12 syncytia after treatment with calyculin A (a myosin II activator) or CN03 (a selective RhoA activator) were visualized by immunofluorescent staining with anti-MyHC antibody and DAPI. Morphologies of the syncytia are shown in Supplementary Figure 6c. Left: cell fusion evaluated as percentages of syncytia containing ≥50 nuclei. Right: polarized elongation evaluated as percentages of syncytia with aspect ratios ≥3. ****P < 0.0001 (Student’s *t*-test). NS not significant, *n* sample number. Bar graphs represent mean ± S.E.M. Box and whiskers graph-line: median, box: upper and lower quartiles, whiskers: maxima and minima. Scale bars: 20 μm (**b**), 100 μm (**d**)

Rho-associated, coiled-coil-containing protein kinase (ROCK) is a primary effector of RhoA GTPase and induces phosphorylation of myosin light chain 2 (MLC2) at the Thr-18 and Ser-19 residues, resulting in NMII activation and subsequent actomyosin assembly^26^. The active forms of RhoA and ROCK were co-localized with F-actin, NMIIA, and MLC2, and partially with PIEZO1, at the lateral cortex of bipolar C2C12 myoblasts (Supplementary Figure 6a). These observations, together with a previous report that the RhoA/ROCK pathway is activated by PIEZO1^42^, suggest that the RhoA/ROCK/actomyosin pathway plays a dominant role in PS flippase-mediated and PIEZO1-mediated myotube formation (Fig. 7a). We found that a phosphorylated form of MLC2 (P-MLC2) had clearly accumulated at the lateral cortex of WT myotubes, while no significant localization was observed in the PIEZO1-deficient, CDC50A-deficient or ATP11A-deficient C2C12 cells (Fig. 7b, c). Stable expression of a phospho-mimetic form of MLC2 (MLC2-DD: T18D, S19D) totally prevented aberrant myotube formation in PIEZO1-deficient C2C12 cells (Fig. 7d, e and Supplementary Figure 6b), indicating that MLC2 phosphorylation is a downstream event of PIEZO1 that leads to actomyosin assembly. Furthermore, both CN03, a selective RhoA activator, and calyculin A, a myosin II activator that inhibits dephosphorylation of MLC2, prevented the myotube formation defects observed with CDC50A-deficient, ATP11A-deficient, and PIEZO1-deficient C2C12 cells (Fig. 7f and Supplementary Figure 6c, d). These results collectively indicate that flippase-mediated PS translocation at the plasma membrane regulates PIEZO1 activation, which promotes RhoA/ROCK-mediated phosphorylation of MLC2 and subsequent assembly of cortical actomyosin fibers, thereby controlling fusion and polarized elongation during myotube formation.

### A role of ATP11A in muscle regeneration

**Fig. 8:**
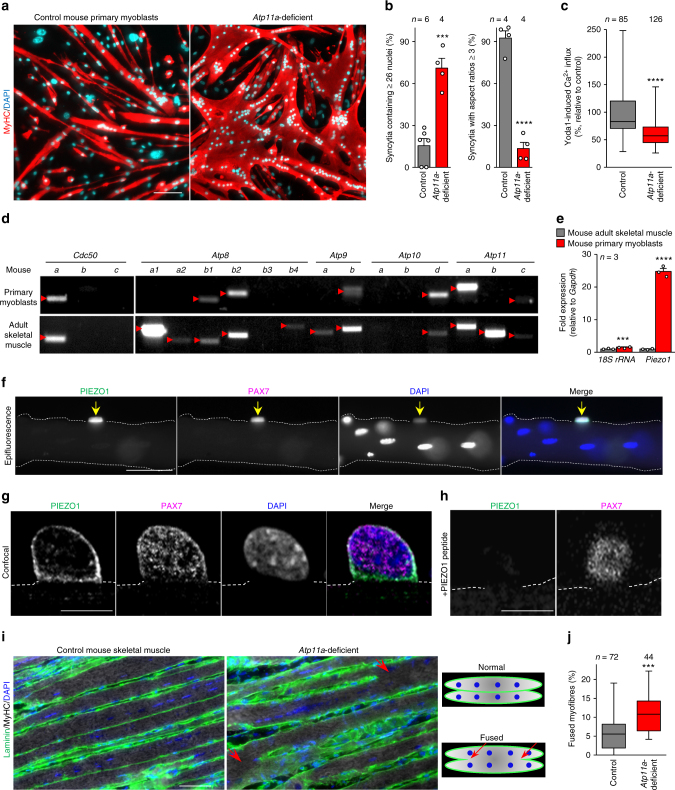
A role of ATP11A in morphogenesis during myofibre regeneration. (**a**, **b**) **a** Aberrant morphologies of *Atp11a*-deficient primary myotubes. Syncytia formed by control or *Atp11a*-deficient mouse primary myoblasts were visualized by immunofluorescent staining with anti-MyHC antibody (differentiated cells, red) and DAPI (nuclei, cyan). **b** Left: cell fusion evaluated as percentages of syncytia containing ≥26 nuclei in **a**. Right: polarized elongation evaluated as percentages of syncytia with aspect ratios ≥3 in **a**. **c** Suppression of agonist-induced PIEZO1 activation in *Atp11a*-deficient mouse primary myoblasts. Quantification of Yoda1-induced Ca^2+^ influx in control or *Atp11a*-deficient mouse primary myoblasts. **d** Semi-quantitative RT-PCR analysis of CDC50 family members and P4-ATPases in mouse primary myoblasts (upper panels) and adult skeletal muscle (lower panels). Arrowheads denote specific bands. **e** Expression levels of *Piezo1* in mouse primary myoblasts and adult skeletal muscle, evaluated by quantitative RT-PCR. *Gapdh* and *18S rRNA* were used as internal controls. **f**–**h** Detection of endogenous PIEZO1 protein in satellite cells. Epifluorescence (**f**) and confocal (**g**, **h**) images of mouse myofibres visualized by immunofluorescent staining with anti-PIEZO1, anti-PAX7 antibodies and DAPI (nuclei). Specificity of the anti-PIEZO1 antibody was confirmed by the decreased fluorescence signal in the sample treated with the epitope peptide (**h**). Arrows indicate Pax7-positive satellite cells. Dashed lines indicate the cell periphery of myofibres. **i**, **j** Aberrant morphologies of *Atp11a*-deficient regenerated myofibres after cardiotoxin-induced muscle degeneration. **i** Longitudinal sections prepared from control (left) and *Atp11a*-deficient adult muscle (right) were probed with anti-laminin antibody (green), anti-MyHC antibody (grey), and DAPI (nuclei, blue). Arrows indicate apparent fusion sites with neighboring myofibres. **j** Evaluation of fused myofibres in **i**. ***P < 0.001 and ****P < 0.0001 (Student’s *t*-test). NS not significant, *n* sample number. Bar graphs represent mean ± S.E.M. Box and whiskers graph-line: median, box: upper and lower quartiles, whiskers: maxima and minima. Scale bars: 100 μm (**a**), 50 μm (**f**, **i**), 10 μm (**g**, **h**)

Since systemic knockout of *Atp11a* results in lethality during embryogenesis^23^, we generated myoblast-specific conditional *Atp11a*-deficient mice utilizing *Myf5*-cre transgenic mice that expressed Cre-recombinase in myoblasts^43^ (Supplementary Figure 7a) and examined the muscle phenotype. Primary myoblasts isolated from the myoblast-specific *Atp11a*-deficient mice formed aberrantly enlarged syncytia upon differentiation (Fig. 8a, b and Supplementary Figure 7b), showing the morphological abnormalities observed with ATP11A-deficient myoblasts (Fig. 1a–d). PIEZO1-mediated Ca^2+^ influx was also attenuated in the *Atp11a*-deficient primary myoblasts (Fig. 8c). However, no obvious morphological or behavioural abnormalities were observed in *Atp11a*-deficient mice by either hematoxylin-eosin staining of cross/longitudinal muscle sections or functional analyses such as grip strength and treadmill running tests. To further evaluate the physiological function of ATP11A and PIEZO1 in myogenesis, we performed detailed analyses on the stage-specific and tissue-specific expression of ATP11A and PIEZO1 in mouse muscle tissues. The expression profile of mRNA encoding P4-ATPases in primary myoblasts isolated from adult skeletal muscle was quite different from that of adult skeletal muscle: among P4-ATPases expressed on the plasma membrane^16^, *Atp11a* was a major flippase in primary myoblasts, while strong expression of *Atp8A1*, *Atp8b2*, and *Atp11a* was observed in adult skeletal muscle (Fig. 8d). In developing muscle^27^, a variety of cell surface P4-ATPases including *Atp8a1*, *Atp8a2*, *Atp8b1*, *Atp8b2*, *Atp9a*, *Atp10d, Atp11a*, and *Atp11c* were expressed^16^ (Supplementary Figure 7c). These results suggest that these P4-ATPases may functionally compensate for the defective expression of ATP11A during developmental myogenesis. Furthermore, no significant expression of PIEZO1 was detected in developing or adult muscle (Fig. 8e and Supplementary Figure 7c). However, we did find robust expression of PIEZO1 in primary myoblasts as well as in Pax7-positive satellite cells, a population of myogenic progenitor cells in adult muscle^44^ (Fig. 8f, g). The fluorescent signal of the PIEZO1 antibody in satellite cells was significantly diminished in the presence of the epitope peptide, confirming the specificity of the antibody (Fig. 8h). In silico analysis of adult regenerating myofibres after muscle injury^45^ showed that expression levels of *Piezo1* as well as *Myog* (a myogenic marker) were significantly upregulated after induction of muscle injury (Supplementary Figure 7d). These observations prompted us to examine the physiological function of ATP11A in muscle regeneration.

Adult skeletal muscle has the ability to efficiently regenerate after different types of injury. Muscle regeneration is mediated by satellite cells residing beneath the basal lamina of muscle fibers, which are activated after injury and undergo myogenic commitment to become fusion-competent myoblasts^44^. The resulting myoblasts fuse with each other to generate nascent syncytia that mature into functional myofibres^10^. In this study, we evaluated the morphology of regenerating myofibres in *Atp11a*-deficient tibialis anterior (TA) muscle. Cardiotoxin, a myotoxic agent that causes degeneration and concomitant regeneration of myofibres^46^, was injected into TA muscle of *Atp11a*-deficient mice. The regenerating muscle tissues were harvested at 2 weeks post-cardiotoxin injection, then morphological analysis was conducted by staining longitudinal sections from the cardiotoxin-injected muscles with anti-laminin antibody (for the extracellular matrix), anti-MyHC antibody (for myofibres), and DAPI (for nuclei). Our immunohistological analyses demonstrated that, upon cardiotoxin administration, abnormal myofibres that fused with each other were evident in cardiotoxin-injected *Atp11a*-deficient TA muscle (Fig. 8i, j), as observed in regenerating muscles of mutant mice that display the in vitro hyperfusion phenotype^47^. These results suggest that ATP11A-mediated PIEZO1 activation plays a crucial role in proper morphogenesis during myofibre regeneration.

## Discussion

The present study provides evidence that the transbilayer redistribution of PS at the plasma membrane, which is mediated by the phospholipid flippase complex of ATP11A and CDC50A, plays a critical role in regulating the Ca^2+^ channel function of the integral membrane protein PIEZO1. Recent studies of structural determination of membrane proteins and molecular simulations of lipid–protein interactions have demonstrated that the structure, function, and dynamics of membrane proteins are significantly affected by associated lipid molecules^48, 49^. However, it remains unclear how lipids act and regulate the function of membrane proteins in biological membranes, because these structural and biophysical studies have been conducted in model systems where membrane proteins are purified and reconstituted in artificial membranes. There is a key difference between artificial and biological membranes; in biological membranes, phospholipids are asymmetrically distributed between the two leaflets of the bilayer, and are actively transported across the bilayer leaflets by the actions of a family of lipid-transport machineries such as phospholipid flippase and scramblase.

Phospholipid flippase catalyzes selective translocation of specific phospholipid species from the outer to the inner leaflet of cellular membranes, thereby generating the asymmetric transbilayer lipid distribution^3, 4, 17, 18^. A locally unbalanced increase in the phospholipid number on one side of the membrane drives bending of the membrane to induce membrane curvature, which supports recruitment of membrane curvature-sensing proteins in vesicle budding within Golgi and endosomal membranes^3, 4^. In addition to affecting the curved structure of membranes, phospholipid flippase is thought to regulate the electrostatic properties of the inner leaflet of the plasma membrane^3^. Phospholipid flippase-mediated local enrichment of PS in the inner leaflet strengthens the electrostatic interaction between the basic domain of cytosolic proteins and the membrane, thereby playing a critical role in the recruitment and activation of various signaling molecules such as small GTPases and those with pleckstrin homology domain^50, 51^. Recent studies have also shown that lateral reorganization of PS to nanocluster formation drives K-Ras assembly on the inner leaflet via interactions between Ras basic residues and negatively charged PS^50^.

Electrostatic interactions of proteins with anionic phospholipids such as PS, phosphatidic acid, and phosphoinositides in the inner leaflet of the plasma membrane^50, 52^ have been shown to play a crucial role in controlling the function of membrane-associated proteins by the so-called ‘electrostatic switch' mechanism^53–57^. Based on the results described here, we propose a lipid-mediated mechanism, namely the ‘flip-flop switch’ mechanism, for tempo-spatial activation of PIEZO1, in which the regulated change in the transbilayer distribution of particular lipids, such as PS, regulates the function of the ion channel.

## Methods

### Reagents

siRNAs and antibodies are summarized in Supplementary Tables 1 and 2, respectively. 1-oleoyl-2-{6-[(7-nitro-2-1,3-benzoxadiazol-4-yl)amino]hexanoyl}-sn-glycero-3-phosphoserine (18:1-06:0, NBD-PS), 1-oleoyl-2-hydroxy-sn-glycero-3-phospho-L-serine (18:1, LysoPS), 1-oleoyl-2-hydroxy-sn-glycero-3-phosphocholine (18:1, LysoPC), 1-oleoyl-2-hydroxy-sn-glycero-3-phosphate (18:1, LysoPA), 1,2-dioleoyl-sn-glycero-3-phosphocholine (18:1, DOPC) and 1,2-dioleoyl-sn-glycero-3-phospho-L-serine (18:1, DOPS) were purchased from Avanti. LysoPS (18:1) analogues were provided by J.A. and T.O.^58, 59^.

### Plasmids

cDNA clone of human PIEZO1 was purchased from Promega. MmCdc50a cDNA was obtained from pCMV-Tag4A-CDC50A^20^. The other mouse cDNA clones were obtained from mouse (C57BL/6J, Japan SLC Inc.) brain or C2C12 myoblasts (ATCC). pcDNA3.1-Clover-mRuby2 (#49089), mPIEZO1-IRES-eGFP (#80925), pX260 (#42229), and pX330 (#42230) were purchased from Addgene. pGFP-AHD was generated according to previous literature^60^.

pX330-PGKpuro was generated by inserting a PGK promoter and a puromycin-resistant gene (from pX260) into pX330. Target sequences were determined using CRISPRdirect^61^ (Supplementary Figures 1d and 4c) and introduced into pX330-PGKpuro. pmClover-N1 and pmClover-C1 were generated by replacing EGFP in pEGFP-N1 and pEGFP-C1 (Clontech) with mClover (monomeric Clover with A206K mutation), respectively. pATP11A-mClover and pmClover-NMIIA were generated by subcloning of ATP11A and NMIIA into pmClover-N1 and pmClover-C1, respectively. pHsPIEZO1-FLAG was made by subcloning HsPIEZO1 into pEGFP-N1, followed by replacing EGFP with a FLAG-tag. pHsPIEZO1-IRES-EGFP was made by inserting HsPIEZO1 into pEGFP-N1, followed by replacing EGFP with the IRES-EGFP sequence obtained from pMmPIEZO1-IRES-EGFP. pHsPIEZO1-Clover was generated by subcloning HsPIEZO1 into pcDNA3.1 (Invitrogen) and inserting Clover at amino acid position 1591 in PIEZO1^37^. pMmPIEZO1Δ838-849-IRES-EGFP was generated by switching from the WT to the deleted sequence between the *Not*I and *Eco*RI sites of pMmPIEZO1-IRES-EGFP. Plasmids for full-length ATP11A, truncated ATP11A (1 bp insertion) and truncated PIEZO1 (13 bp deletion) were generated by subcloning the corresponding cDNA fragments with a C-terminal FLAG tag into pIRES2-Venus. pMLC2-WT-mClover-P2A-puro was constructed by subcloning MLC2 into pEGFP-N1, followed by replacing EGFP with the gene cassette composed of mClover, a self-cleavage P2A sequence and a puromycin-resistant gene (from pX260). pMLC2-DD-mClover-P2A-puro was constructed by introducing phospho-mimetic mutations (T18D/S19D) into pMLC2-WT-mClover-P2A-puro. pCDC50A and pCDC50A-FLAG were generated by subcloning CDC50A into pCMV-Tag4A (Agilent Technologies) with and without a stop codon, respectively. pCA-TMEM16F-mRuby2 was generated by subcloning the cDNA encoding CA-TMEM16F^39^ into pEGFP-N1 and replacing EGFP with mRuby2. pEGFP-P4-ATPases and pEGFP-AP3D1 were generated by subcloning the corresponding full-length into pEGFP-C1. Plasmids of mechanosensitive channels (pMSCs) were generated by inserting the qPCR-targeting fragment into pMD-20 (Takara). Retroviral vectors expressing P4-ATPases or CDC50A were generated by subcloning the corresponding full-length with FLAG- or mClover-tag at the C-terminus into pMXs-puro (Dr. Toshio Kitamura, the University of Tokyo). Plasmids were confirmed by sequencing.

### Cells

C2C12, HEK293 cells, and human primary myoblasts were purchased from ATCC, RIKEN BRC and Thermo, respectively. Plat-E cells were a gift from Dr. Toshio Kitamura. Cells were grown in 10–20% FBS/DMEM in a 37 °C incubator with 5% CO_2_.

For CRISPR/Cas9-mediated gene editing^28^, C2C12 cells were transfected with pX330-PGKpuro using ViaFect (Promega) for 1 day and cultured with puromycin (1–3 μg/mL) for 3–5 days. Survived cells were cloned by limited dilution and genotyped (Supplementary Figures 1d and 4c). Data about CDC50A-deficient, ATP11A-deficient, and PIEZO1-deficient C2C12 cells were obtained from the corresponding clone #1 unless otherwise noted.

For stable lines expressing MLC2, C2C12 cells were transfected with pMLC2-WT-mClover-P2A-puro or pMLC2-DD-mClover-P2A-puro using CUY21EDITII electroporator (BEX) and cultured with puromycin. For stable lines expressing P4-ATPases or CDC50A, Plat-E cells were transfected with the pMXs-puro vectors using Viafect. Resultant retroviruses were used to infect C2C12 cells. Two days after infection, the cells were cultured with puromycin.

### Myotube formation

C2C12 cells or primary myoblasts confluently grown in 20% FBS/DMEM were maintained in differentiation media [DMEM containing 2% horse serum (Gibco)] for the indicated periods. For RNA interference, cells were transfected twice with siRNA using Lipofectamine RNAiMAX (Thermo) and maintained in differentiation medium for 3 days. In pharmacological experiments, cells cultured in differentiation media for 2 days were incubated with calyculin A (1 nM, Wako) or CN03 (2 μg/mL, Cytoskeleton) for an additional 2 days. Z-VAD-FMK (50 µM, Peptide institute) was added to cells 1 day before switching to differentiation medium and maintained for 4 days.

Four days after differentiation, myotubes were stained with anti-MyHC antibody (14-6503-82, dilution 1:500, eBioscience) and DAPI (Dojindo), and visualized with an epifluorescence microscope (Axio-Observer.Z1, Zeiss) with a 10× objective lens. The number of nuclei, cell area and best-fit ellipse aspect ratio were calculated using ImageJ software. Cell fusion was evaluated as the percentage of nuclei in multinucleate MyHC^+^ cells (containing ≥16 nuclei) versus the total number of nuclei in MyHC^+^ cells in each microscopic field^27^. Polarized elongation was evaluated as percentages of the sum area occupied by MyHC^+^ cells with ≥3 aspect ratios versus the total area of MyHC^+^ cells in the field^62, 63^. Differentiation level was evaluated as percentages of nuclei in MyHC^+^ cells versus the total number of nuclei in each microscopic field^27^.

### RT-PCR

Total RNA was isolated using ISOGEN II (Nippon Gene) and NucleoSpin RNA (Macherey-Nagel). cDNA was generated with PrimeScript II RTase (Takara) or ReverTra Ace qPCR RT Master Mix (Toyobo). Semi-qPCR was performed with EmeraldAmp MAX PCR Master Mix (Takara). qPCR was performed with PowerUp SYBR Green Master Mix (Thermo) using the StepOne system (Thermo). Copy numbers were determined using standard curves from pEGFP-P4-ATPases, pMSCs and pEGFP-AP3D1, and compared to *Ap3d1*^64^. Relative expression was calculated using the 2^−ΔΔCt^ method. Primers are listed in Supplementary Tables 3 and 4.

### Co-immunoprecipitation and immunoblotting

For co-immunoprecipitation, HEK293 cells were transfected with pEGFP-P4-ATPases and pCDC50A-FLAG using X-tremeGENE HP DNA (Roche). Two days after transfection, the cells were lysed in cold buffer (150 mM NaCl, and 50 mM Tris at pH 7.4 containing 1% Triton X-100 and protease inhibitors). After removal of insoluble fractions by centrifugation, the supernatants were incubated with anti-FLAG M2 affinity gel (Sigma) for 30 min on ice. After washing with 0.1% Triton X-100/TBS, immunoprecipitates were eluted with FLAG peptides (500 μg/mL, Sigma) for 30 min on ice. Resulting supernatants were incubated in SDS sample buffer at 50 °C for 5 min.

For immunoblotting, proteins were separated by SDS-PAGE and transferred to polyvinylidene difluoride membrane. The membrane was blocked in TBS containing 5% skim milk and 0.1% Tween 20, stained with antibodies and visualized using an enhanced chemiluminescent reagent. Uncropped immunoblots are shown in Supplementary Figure 8.

### Immunofluorescent analyses

Cells were fixed with 4% paraformaldehyde (PFA)/PBS, permeabilized in 0.1% Triton X-100/PBS, blocked in 1–2% BSA/PBS and probed with antibodies. An anti-goat tertiary antibody (A-11055, dilution 1:500, Thermo) was used to detect P-MLC2. Optical sections in the middle of cells were obtained using a confocal microscope (LSM710, Zeiss) with a 63× objective lens. Fluorescence profiles were analyzed using Zen software (Zeiss).

To determine PIEZO1 localization, either pHsPIEZO1-Clover or pHsPIEZO1-FLAG alone, pHsPIEZO1-FLAG together with pGFP-AHD, pMLC2-WT-mClover-P2A-puro or pmClover-NMIIA (1:1), or pHsPIEZO1-FLAG/pATP11A-mClover/pCDC50A (1:1:1) were introduced into C2C12 myoblasts by electroporation. Cells were cultured on μ-Dishes ibiTreat for 1 day and probed with phalloidin-TRITC (Sigma), anti-FLAG (F1804, dilution 1:500, Sigma), anti-GFP (598, dilution 1:1000, MBL), and anti-ROCK1 (GTX113266, dilution 1:100, GeneTex) antibodies.

To analyze NMIIA or P-MLC2 localization^33^, C2C12 cells on μ-Dishes ibiTreat were placed in differentiation medium for 3 days and stained with phalloidin-TRITC, DAPI, anti-NMIIA (M8064, dilution 1:50, Sigma), and anti-P-MLC2 (3671, dilution 1:50, CST) antibodies. Cell edges were divided into multiple rectangles orthogonal to the cell periphery. Within the fluorescence profile in each rectangle, the peak intensity between 0 and 5 μm from the cell edge and the mean intensity between 2 and 7 μm from the peak position were calculated. The cortex/cytoplasm ratio was determined as the average of the peak versus the mean intensity of all rectangles.

### Time-lapse imaging

C2C12 cells on μ-Dishes ibiTreat were placed in differentiation medium for 2 days and visualized with an LCV110 incubator microscope equipped with a 20× objective lens (Olympus). DIC images were captured every 20 min for 2 days.

### NBD-PS flipping assay

C2C12 myoblasts were detached by Cell Dissociation Solution Non-enzymatic 1× (Sigma), harvested by centrifugation and washed with HBSS (Thermo). After being placed on ice for at least 10 min, the assay was initiated by incubation with 0.1 μM NBD-PS for the indicated periods at 15 °C. The reaction was terminated by resuspension of the cells in HBSS containing fatty-acid-free BSA (3 mg/mL, Sigma). Cells (0.5–1 × 10^4^) were analyzed with a flow cytometer (Cytomics FC 500, Beckman). Median fluorescence intensities of cell populations were determined using FlowJo software (Tree Star).

### Phospholipid exchange

C2C12 cells trypsinized for annexin V labelling or grown on coverslips for Ca^2+^ measurements were incubated in PBS or DMEM containing 4 mM MαCD (2,6-Di-*O*-methyl-α-cyclodextrin, Wako) and 0.3 mM DOPC or 3:1 (mol:mol) DOPC/DOPS for 20 min at 37 °C^40^.

### Annexin V labelling

For flow cytometry, C2C12 cells were detached, re-suspended in binding buffer (140 mM NaCl, 2.5 mM CaCl_2_ and 10 mM HEPES at pH 7.4) and incubated with annexin V-EGFP (Promokine) at room temperature (RT) for 5 min. Cells (2–4 × 10^4^) were analyzed with a flow cytometer (Cytomics FC 500, Beckman). Histograms were drawn using FlowJo. Gating strategies are shown in Supplementary Figure 8.

For confocal microscopy, human primary myoblasts were differentiated on μ-Dishes ibiTreat for 2 days. After washing with binding buffer, the cells were incubated with Fixable Viability Dye eFluor 660 (Thermo) at RT for 15 min. Cells were treated with annexin V-EGFP at RT for 5 min, fixed with 4% PFA/PBS at RT for 10 min and stained with DAPI.

For Ca^2+^ measurements, human primary myoblasts were differentiated on Cell Desk LF1 (Sumitomo Bakelite) for 2 days, incubated with Fura2-AM (15 μM, Dojindo) at RT for 30 min, washed with binding buffer and incubated with annexin V-EGFP at RT for 5 min.

### F2N12S measurements

C2C12 cells were detached, suspended in HBSS and placed on ice for at least 10 min. Then, the cells (0.1−1 × 10^7^ cells/mL) were incubated with F2N12S (0.1 μM, Thermo) for 5 min at 15 °C. Emission spectra were recorded at an excitation wavelength of 400 nm using an LS55 Fluorescence Spectrometer (Perkin Elmer) at RT. Spectra were averaged over three replicates and corrected for lamp intensity variations and blanks.

### Ca^2+^ measurements

Ca^2+^ measurements were conducted according to previous literature^65^. HEK293 cells were transfected with pMmPIEZO1-IRES-EGFP and pCA-TMEM16F-mRuby2 using ViaFect. The cells were then seeded onto poly-L-lysine-coated coverslips at one day post transfection and cultured overnight. C2C12 cells on poly-L-lysine-coated coverslips were transfected with plasmids expressing PIEZO1 or ATP11A and cultured overnight. Human primary myoblasts on poly-L-lysine-coated coverslips were transfected with siRNA and cultured for 2 days. Cells were loaded with Fura2-AM (5 μM) in 10% FBS/DMEM at 37 °C for 40 min and washed with HEPES-buffered saline (HBS)^65^. The coverslip was placed in a perfusion chamber mounted on a microscope (Axio-observer Z1) at RT. Time-lapse images were recorded every 10 s. Transfected cells were identified by fluorescent proteins. Cells were perfused with HBS for 2 min, then the perfusion solution was replaced with HBS containing Yoda1 (0.3–10 μM, Maybridge or Tocris) for 8 min. To analyze the effect of lyso-lipids, C2C12 cells were perfused with HBS containing 1–3 μM lyso-lipid for 5 min, then the perfusion solution was changed to 1 μM Yoda1/HBS for 8 min. After treatment with 3 μM LysoPS for 5 min, the perfusion solution was changed to HBS containing fatty-acid-free BSA (5 mg/mL) for 5 min, followed by 1 μM Yoda1/HBS for 8 min. To analyze the effect of LysoPS analogues, cells were incubated with 3 μM LysoPS analogues for 5 min, perfused with HBS for 2 min, then the perfusion solution was changed to 1 μM Yoda1/HBS for 10 min. Ratiometric images (F_340_/F_380_) were analyzed with Physiology software (Zeiss). Yoda1-induced Ca^2+^ influx was quantified as the difference in the Fura2 ratio between its maximum value and that at 1 min from imaging initiation.

### Electrophysiology

HEK293T cells were transfected with pMmPIEZO1-IRES-EGFP using Lipofectamine 2000 (Thermo) and reseeded onto coverslips. Whole-cell recordings on HEK293T expressing MmPIEZO1 at 24–36 h post-transfection were conducted according to previous literature^24^. The internal solution contained (in mM): 140 CsCl, 5 EGTA, 10 HEPES, pH = 7.4 adjusted with CsOH, and the standard bath solution contained (in mM): 140 NaCl, 5 KCl, 2 CaCl_2_, 2 MgCl_2_, 10 glucose, and 10 HEPES, pH = 7.4 adjusted with NaOH). The folding potential was −60 mV and currents were recorded at a whole-cell configuration using Axopatch 200B amplifier (Molecular Devices), filtered at 5 kHz with a low-pass filter and digitized with Digidata 1440 A (Axon Instruments). Data were acquired with pCLAMP 10 (Axon Instruments). Glass pipettes (8250; King Precision Glass Inc.) had a resistance of 2–4 MΩ. Mechanical stimulation was provided by membrane indentation (8 μm) with a glass pipette for 150 ms. Currents were recorded before (control) and after administration of 5 μM lyso-phospholipid for 1 min, and after washing with fatty-acid-free BSA for 1 min.

Cell-attached recordings were carried out according to previous literature^66^. Electrophysiological recordings on MmPIEZO1-expressing HEK293 cells at 36–60 h post-transfection were conducted using an EPC10 amplifier and Patchmaster software (HEKA Elektronik). Data were acquired at 5 kHz and filtered at 2.9 kHz. Glass pipettes (1.5 OD, 0.85 ID; Sutter Instrument Company) had a resistance of 1.5–4 MΩ. The internal and bath solutions were prepared according to previous literature^66^. Pressure was administrated using a syringe while monitoring the pressure with a manometer. Patches were held at −60 mV and stimulated with negative pressure for 2 s. LysoPS (3 μM) was added to the internal solution.

### Membrane tension measurements

An optical trap system (MMS-1064–200–2L/2E/2S, Sigma-koki) with a 1064-nm laser was used to trap cell-attached beads. A 100× oil objective (UPLSAPO100XO, NA = 1.4, Olympus) mounted on an inverted microscope (IX71, Olympus) was used to trap beads and visualize cells and beads in bright field. A motorized stage (BIOS-225T, Sigma-koki) controlled by a custom LabView program (National Instruments) was used to pull cells away from the stationary laser trap.

Trap stiffness calibration was performed by measuring the position variance of a trapped bead undergoing Brownian motion—a passive stiffness calibration based on equipartition theorem, assuming the thermal energy for each degree of freedom to be *k*_B_*T*/2 (where *k*_B_ is the Boltzmann constant and *T* the absolute temperature). The particle is trapped in a harmonic potential in the *x*-direction,

1 $$V_x = k_x\left\langle {x^2} \right\rangle /2.$$

*k*_*x*_ is the trap stiffness and 〈*x*^2^〉 the position variance of the trapped particle. Using the information of the trapped particle coordinates:

2 $$k_{x} = k_{\mathrm{B}}T/\left\langle x \right\rangle ^{2}$$

The same applies for fluctuation in the *y*-axis direction.

Images of a trapped bead were captured at 1000 frames/s for 10 s, with the exposure time of the CCD camera (Zyla 5.5 cMOS, Andor) set to 1 ms. The center of mass coordinates of the trapped bead was obtained using ImageJ software. Variance in the *x*-axis direction 〈*x*^2^〉 was obtained by a Gaussian fit to the probability distribution of *x*. This resulted in a spring constant *k*_*x*_ = 7.29 ± 0.50 [pN/μm]. We also confirmed that *k*_*y*_ was similar to that of *k*_*x*_.

For tether pulling experiments, C2C12 cells on poly-L-lysine-coated glass bottom dishes (Matsunami) were incubated in 10% FBS/DMEM containing streptavidin-coated polystyrene beads (1.76 μm diameter, Spherotech) conjugated with biotinylated concanavalin A (J-OIL MILLS) for 1–2 h at 37 °C. For actin depolymerization, cells were incubated with cytochalasin D (20 μM, Sigma) for 2 h and washed with 10% FBS/DMEM. Cell-attached beads were trapped and pulled by moving the stage at 1 μm/s. Images were captured with a CCD camera (Zyla 5.5 cMOS, Andor) every 10 ms.

To obtain membrane tension, force-extension curves were acquired by calculating the force *F* exerted on the trapped bead as

3 $${{F}} = k_{{x}}{\mathrm{\Delta }}x,$$

where Δ*x* is the deviation of the trapped bead from its original position. A linear fit was applied to the first linear part of the force-extension curves.

### PS content

PS was separated from total lipid by two-dimensional thin-layer chromatography with the first solvent system of chloroform/methanol/acetic acid (65:25:10, v/v/v) and the second solvent system of chloroform/methanol/formic acid (65:25:10, v/v/v) on a silica plate and assessed by inorganic phosphate quantification^67^.

### Analysis of *Atp11a*-deficient mice

Animal care, ethical usage and procedures were approved by the Animal Care Use and Review Committee of the Department of Engineering of Kyoto University. A transgenic mouse strain (*Atp11a*^*tm1a(KOMP)Wtsi*^) harboring the ‘knockout-first’ conditional cassette^68^ in the *Atp11a* gene was purchased from EMMA. *Atp11a*^*tm1a(KOMP)Wtsi*^ was crossed with B6-Tg(CAG-FLPe)36 mice^69^ to generate *Atp11a*^*tm1c(KOMP)Wtsi*^ mice. The resultant mice were further mated with *Myf5*-cre transgenic mice on a C57BL/6 background^43^, to generate myoblast-specific *Atp11a-*deficient mice.

Myoblasts were isolated from extensor digitorum longus (EDL) muscles according to previous literature^70^. EDL myofibres were fixed with 4% PFA/PBS, permeabilized in 0.5% Triton X-100/PBS and blocked in 3% BSA/PBS. Samples were stained with anti-Pax7 and anti-PIEZO1 antibodies in the presence or absence of PIEZO1 peptide (5 μg/mL, Novus Biologicals) and visualized with a confocal microscope (LSM-800, Zeiss) or an epifluorescence microscope (Axio-observer Z1).

Cardiotoxin experiments were carried out according to previous literature^46^. Fifty microliters of 10 μM cardiotoxin (Sigma or Latoxan) was injected into tibialis anterior muscle of 8- to 12-week-old mice. The muscle was harvested at 2 weeks post-injection, and snap-frozen in isopentane cooled with liquid nitrogen. Longitudinal cryosections (thickness, 7 µm) obtained from the muscle were stained with anti-MyHC and anti-laminin antibodies and visualized with a microscope (Axio-observer Z1). In silico analysis on regenerating muscle was conducted by using NCBI dataset (#GDS4924)^45^.

### Statistics and reproducibility

Representative figures are shown as data from a single experiment of multiple experiments or average data from pools across multiple experiments. Samples in quantitative data indicate microscopic fields (morphological analysis), single cells (flow cytometry, localization analysis, Ca^2+^ imaging, membrane tension measurement, electrophysiology), cell cultures (RT-PCR, PS quantification, F2N12S measurement) and muscles (in silico analysis). Quantitative data of single-cell-based or microscopic analyses were derived from pools across multiple experiments. All tests were repeated at least three times. Statistical significance was determined with the paired Student’s *t*-test. Bar and box-whiskers graphs, sample size (*n*) and *P* values are specified in the figure legends. No statistical methods to pre-specify sample size were used. No randomization was used. No blinding was done.

### Data availability

Data supporting the findings of this manuscript are available from the corresponding authors upon reasonable request.

## Electronic supplementary material

Supplementary Information

Peer Review File

Description of Additional Supplementary Information

Supplementary Movie 1

Supplementary Movie 2

Supplementary Movie 3

Supplementary Movie 4
