# Supplementary material for: Cell surface flip-flop of phosphatidylserine is critical for PIEZO1-mediated myotube formation
Source: Nat Commun. 2018 May 24;9:2049. doi: 10.1038/s41467-018-04436-w (PMC5967302; doi:10.1038/s41467-018-04436-w)
Supplement: Supplementary file 1 — Supplementary Information [file 41467_2018_4436_MOESM1_ESM.pdf]

Supplementary Information

Cell surface flip-flop of phosphatidylserine is critical for PIEZO1-mediated myotube formation

Tsuchiya et al.

Supplementary Figures 1-8

Supplementary Tables 1-4

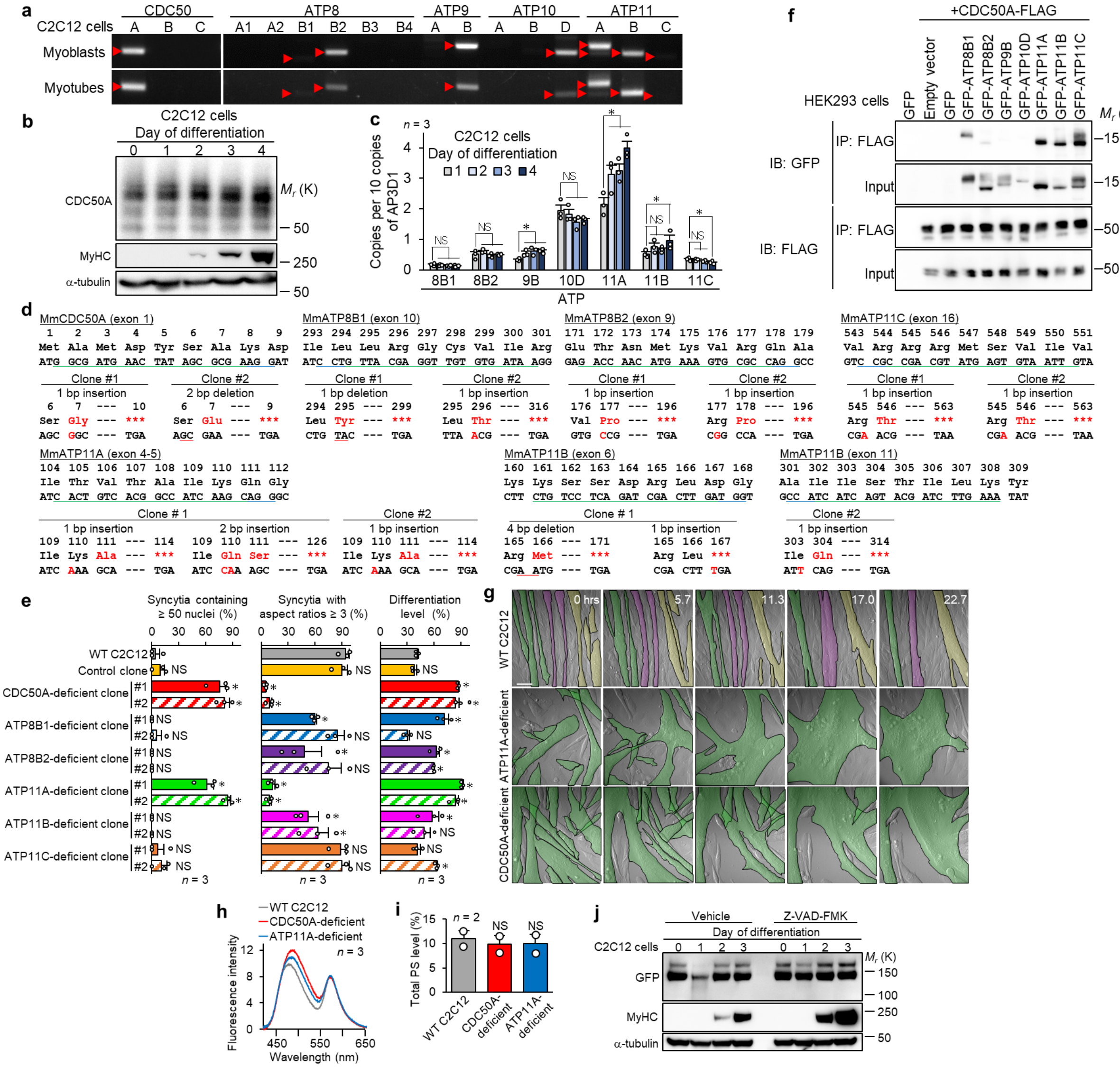

Supplementary Figure 1. Supporting data for defective myotube formation by CDC50A- and ATP11A-deficient C2C12 myoblasts. (a-c) Expression of phospholipid flippase complex components in C2C12 cells. (**a**) Semi-quantitative RT-PCR analysis of CDC50 family members and P4-ATPases in proliferating myoblasts and differentiated myotubes from C2C12 cells. Red arrowheads denote specific bands. (**b**) Immunoblot analysis of CDC50A in differentiated C2C12 cells. MyHC and α-tubulin were detected as a differentiation marker and a loading control, respectively. (**c**) Quantitative RT-PCR analysis of P4-ATPases (compared to AP3D1) in differentiated C2C12 cells. **(d-g)** Defective myotube formation by C2C12 myoblasts deficient in the PS flippase complex of ATP11A and CDC50A. (**d**) CRISPR/Cas9 target sites in the indicated genes of C2C12 cells. The guide sequence and the protospacer-adjacent motif in each gene are underlined in green and blue, respectively. CRISPR/Cas9-introduced insertions or deletions are shown in red characters. (**e**) Characterization of myotube formation in control and flippase-deficient C2C12 clones by immunofluorescence imaging of MyHC. Quantification of cell fusion (left), polarized elongation (middle) and differentiation (right). (**f**) Immunoblot analysis (IB) of CDC50A-associated P4-ATPases in anti-FLAG immunoprecipitates (IP) of HEK293 cells co-expressing FLAG-tagged CDC50A and one of the GFP-tagged P4-ATPases visualized with anti-FLAG and anti-GFP antibodies. (**g**) Time-lapse images of myotube formation in WT, CDC50A- and ATP11A-deficient C2C12 cells, began at 2 days (0 hrs) after induction of differentiation. These images were selected from Supplementary Movies 1-3. Syncytia are pseudo-coloured and the cell periphery is indicated by a black line. (**h**) Increased anionic phospholipids on the cell surface of PS flippase-deficient myoblasts. Fluorescent spectra of F2N12S (a membrane asymmetry probe sensitive to anionic phospholipids in the plasma membrane outer leaflet) in WT, CDC50A- and ATP11A-deficient C2C12 myoblasts. (**i**) Normal PS content in PS flippase-deficient myoblasts. Thin-layer chromatography analysis of total PS levels in WT, CDC50A- and ATP11A-deficient C2C12 myoblasts. (**j**) Transient ATP11A degradation by caspases during myotube formation. Immunoblot analysis of differentiated C2C12 cells stably expressing ATP11A-GFP in the presence or absence of Z-VAD-FMK using anti-GFP, anti-MyHC and anti-α-tubulin antibodies. *$P < 0.05$ (Student's t-test). NS, not significant. *n*, sample number. Error bars represent the S.E.M. Scale bar, 50 μm (g).

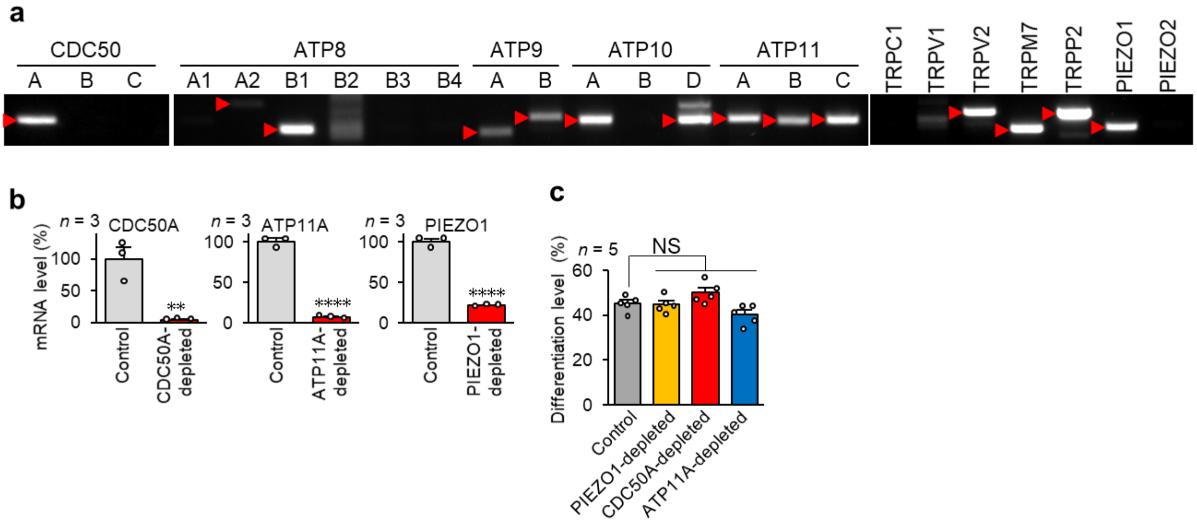

Supplementary Figure 2. Supporting data for defective myotube formation by PS flippase- and PIEZO1-depleted human primary myoblasts. (**a**) Semi-quantitative RT-PCR analysis of CDC50 family members, P4-ATPases and a series of Ca^{2+}-permeable mechanosensitive channels in human primary myoblasts. Red arrowheads denote specific bands. (**b**) Quantitative RT-PCR analysis of PIEZO1, CDC50A and ATP11A in human primary myoblasts transfected with non-targeting negative control siRNA or siRNA against the corresponding genes. *GAPDH* was used as the reference gene. (**c**) Quantification of differentiation in Fig. 1c and 2c. $^{**}P < 0.01$ and $^{****}P < 0.0001$ (Student's t-test). NS, not significant. *n*, sample number. Error bars represent the S.E.M.

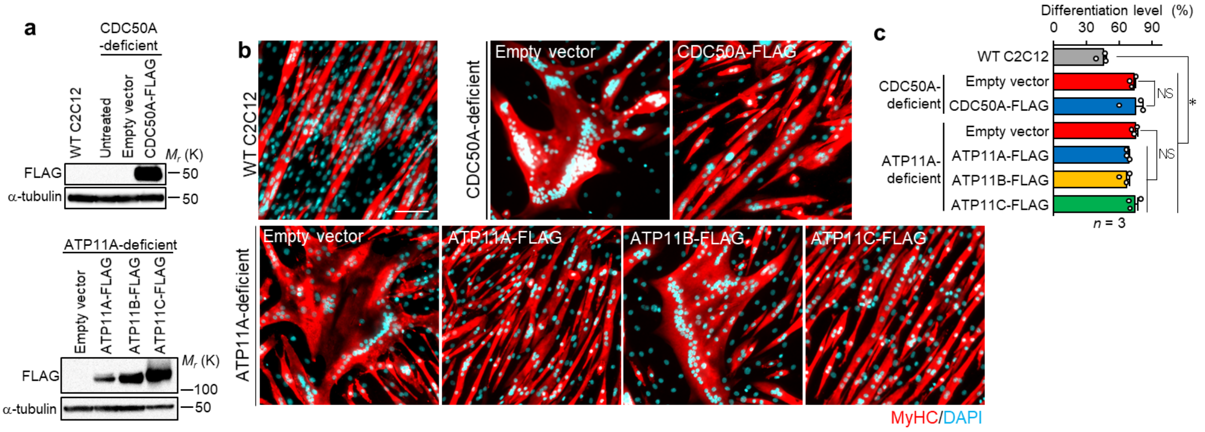

Supplementary Figure 3. Supporting data for rescue of morphologies in PS flippase-deficient C2C12 myotubes by overexpression of PS flippase complex components. (**a**) Immunoblot analysis of WT, CDC50A- and ATP11A-deficient C2C12 cells infected with retroviruses expressing FLAG-tagged proteins. (**b**) Syncytia formed by WT, CDC50A- or ATP11A-deficient C2C12 myoblasts expressing FLAG-tagged proteins were visualized by immunofluorescent staining with anti-MyHC antibody (differentiated cells, red) and DAPI (nuclei, cyan). (**c**) Quantification of differentiation in **b**. *$P < 0.05$ (Student's t-test). NS, not significant. n, sample number. Error bars represent the S.E.M. Scale bar, 100 μm (b).

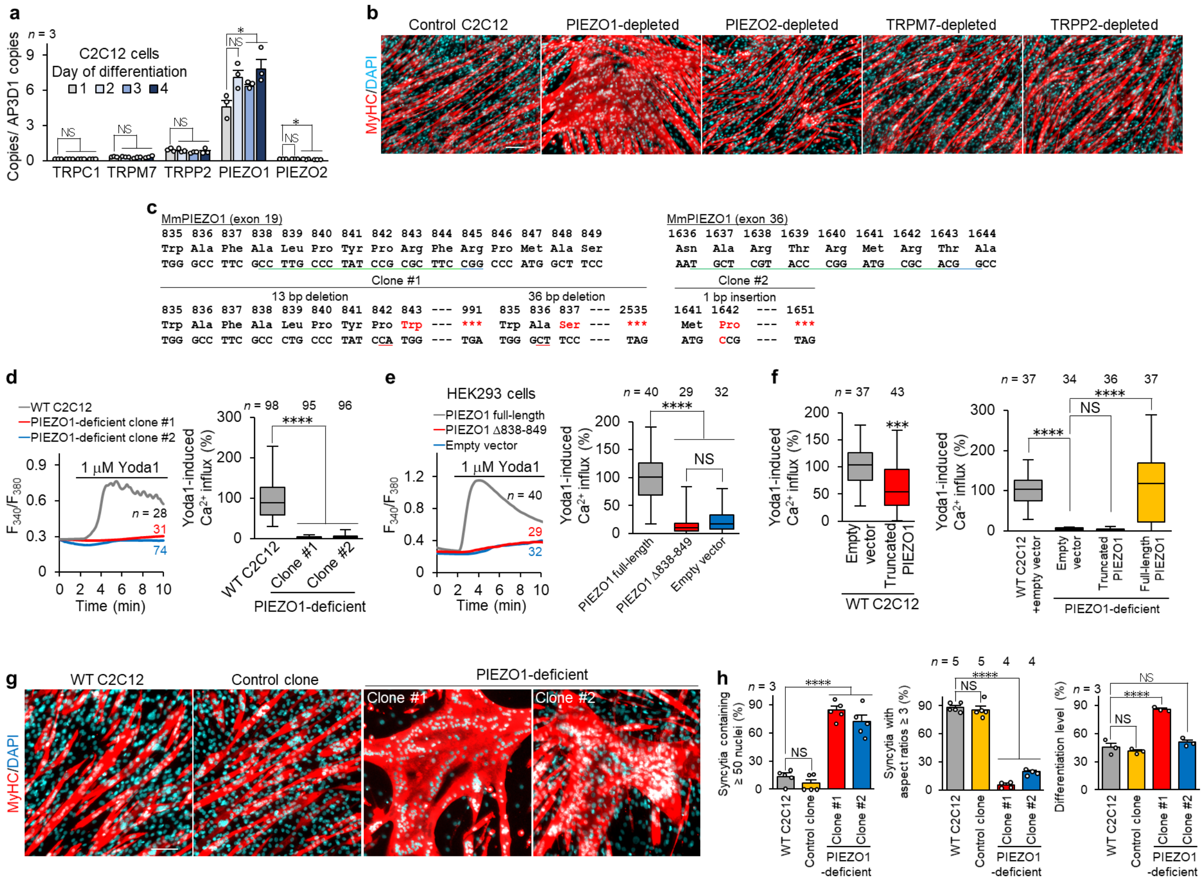

Supplementary Figure 4. Supporting data for defective myotube formation by PIEZO1-deficient C2C12 myoblasts. (**a**) Quantitative RT-PCR analysis of the Ca^{2+}-permeable mechanosensitive channels (compared to AP3D1) in differentiated C2C12 cells. (**b**) Defective myotube formation by PIEZO1-depleted C2C12 myoblasts. Morphologies of C2C12 myotubes transfected with siRNA against PIEZO1, PIEZO2, TRPM7 and TRPP2, stained for MyHC and nuclei. (**c**) CRISPR/Cas9 target sites in the PIEZO1 gene of C2C12 cells. The guide sequence and the protospacer-adjacent motif are underlined in green and blue, respectively. CRISPR/Cas9-induced insertions or deletions are shown in red characters. (**d**) Suppression of Yoda1-induced Ca^{2+} influx in PIEZO1-deficient C2C12 clones. Representative traces (left) and quantification (right) of Yoda1-induced Ca^{2+} influx in WT C2C12 cells and PIEZO1-deficient clones. (**e, f**) Impaired channel function of the PIEZO1 mutants identified in the PIEZO1-deficient C2C12 clones. (**e**) Representative traces (left) and quantification (right) of Yoda1-induced Ca^{2+} influx in HEK293 cells transfected with PIEZO1 (full-length or Δ838-849). (**f**) Left: Quantification of Yoda1-induced Ca^{2+} influx in WT C2C12 cells transfected with an empty vector or truncated PIEZO1 (13 bp deletion). Right: Quantification of Yoda1-induced Ca^{2+} influx in WT and the PIEZO1-deficient C2C12 cells transfected with truncated PIEZO1 (13 bp deletion). (**g, h**) Aberrant myotube morphologies in PIEZO1-deficient C2C12 clones. (**g**) Syncytia formed by WT C2C12 cells, control clones and PIEZO1-deficient clones were visualized by anti-MyHC antibody (differentiated cells, red) and DAPI (nuclei, cyan). (**h**) Quantification of cell fusion (left), polarized elongation (middle) and differentiation (right) in **g**. *$P < 0.05$, ***$P < 0.001$ and ****$P < 0.0001$ (Student's *t*-test). NS, not significant. *n*, sample number. Bar graphs represent mean ± S.E.M. Box and whiskers graph—line: median, box: upper and lower quartiles, whiskers: maxima and minima. Scale bars: 100 µm (b, g).

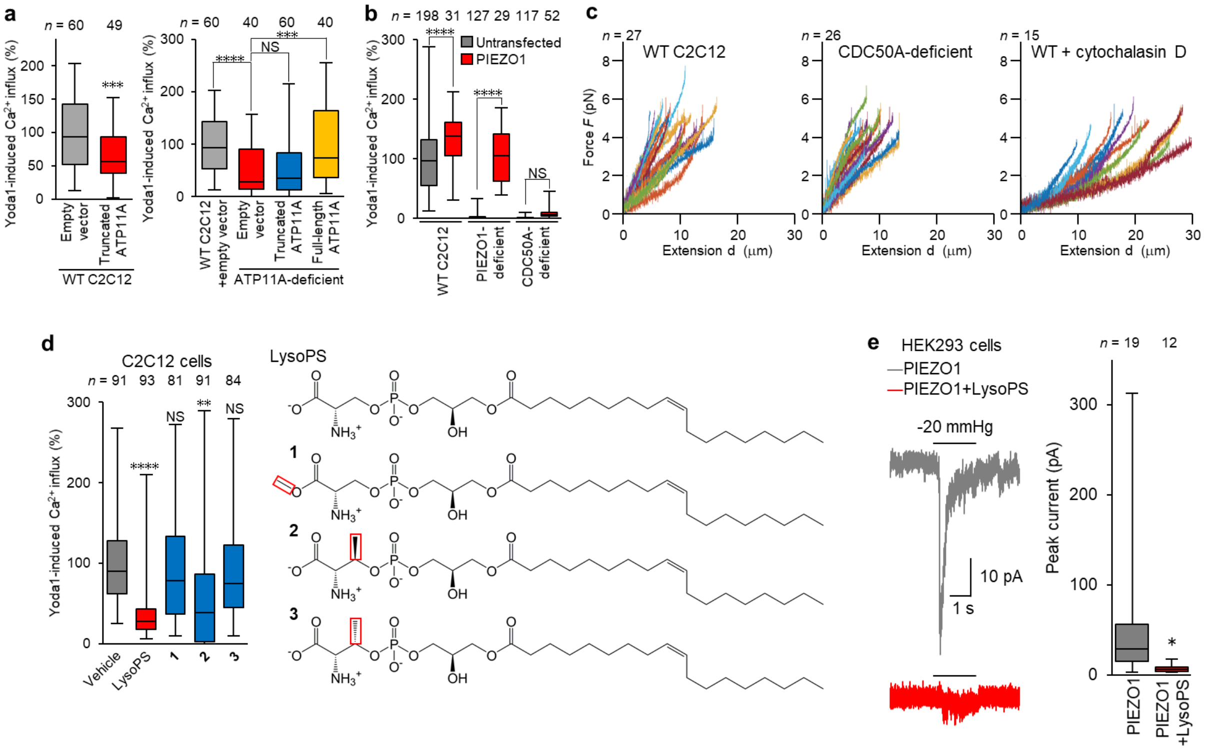

Supplementary Figure 5. Supporting data for suppression of PIEZO1 activation by PS flippase deficiency and cell surface-inserted LysoPS. (**a**) Dysfunction of the ATP11A mutant identified in the ATP11A-deficient C2C12 clones. Quantification of Yoda1-induced Ca^{2+} influx in WT (left) or the ATP11A-deficient (right) C2C12 cells expressing truncated ATP11A (1 bp insertion). (**b**) Failed rescue of impaired PIEZO1 activation in PS flippase-deficient myoblasts by overexpression of PIEZO1. Quantification of Yoda1-induced Ca^{2+} influx in WT, the PIEZO1-deficient and CDC50A-deficient C2C12 cells expressing PIEZO1. (**c**) Force-extension curves for WT, CDC50A-deficient and cytochalasin D-treated WT C2C12 cells. Membrane tension is obtained by linear fitting of the first linear part of the force-extension curve. (**d**) Stereospecific inhibition of agonist-induced PIEZO1 activation by the phosphoserine headgroup of cell surface-inserted LysoPS. Quantification (left) of Yoda1-induced Ca^{2+} influx in WT C2C12 cells treated with LysoPS analogues **1**-**3** (right). (**e**) Inhibition of negative suction-induced PIEZO1 activation by cell surface-inserted LysoPS. Traces (left) and quantification (right) of currents recorded with negative pipette pressure (–20 mm Hg) from PIEZO1-expressing HEK293 cells in the presence of LysoPS. *$P < 0.05$, **$P < 0.01$, ***$P < 0.001$ and ****$P < 0.0001$ (Student's *t*-test). NS, not significant. *n*, sample number. Box and whiskers graph—line: median, box: upper and lower quartiles, whiskers: maxima and minima.

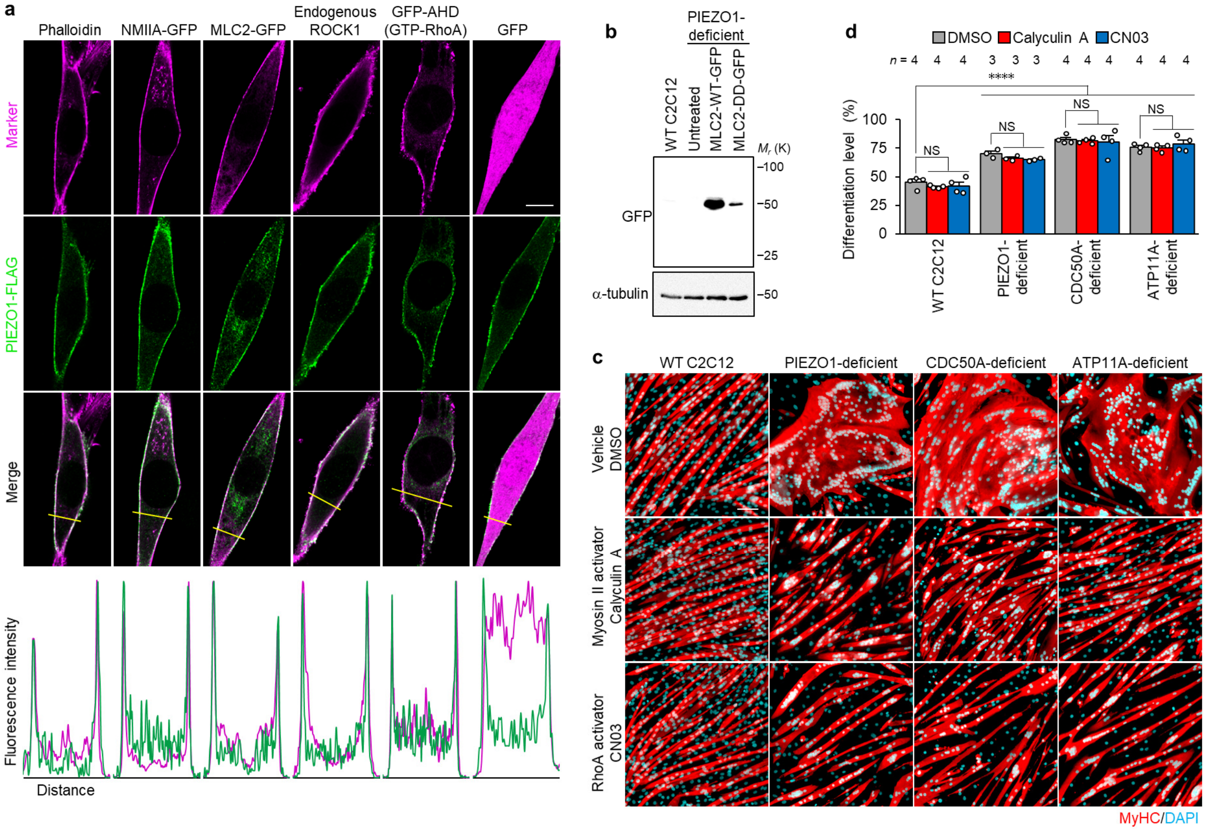

Supplementary Figure 6. Supporting data for RhoA/ROCK-mediated actomyosin formation via the PS flippase/PIEZO1 pathway. (**a**) Colocalization of PIEZO1 with F-actin, NMIIA, MLC2, ROCK1 and active RhoA at the cell cortex of bipolar C2C12 myoblasts. Immunofluorescent analysis of WT C2C12 cells expressing PIEZO1-FLAG alone or with NMIIA-GFP, MLC2-GFP, GFP-AHD or GFP, visualized by phalloidin, anti-FLAG, anti-GFP and anti-ROCK1 antibodies. Fluorescence profiles at yellow lines are shown at the bottom. (**b**) Immunoblot analysis of WT C2C12 cells and PIEZO1-deficient cells untreated or stably expressing MLC2-WT-GFP or MLC2-DD-GFP, visualized by anti-GFP and anti-α-tubulin antibodies. (**c**) Rescue of myotube formation by activation of the RhoA/ROCK/actomyosin pathway in PS flippase- or PIEZO1-deficient C2C12 syncytia. Morphology of myotubes formed by WT, PIEZO1-, CDC50A- and ATP11A-deficient C2C12 cells treated with DMSO, calyculin A and CN03, stained for MyHC and nuclei. (**d**) Quantification of differentiation in **c**. ****$P < 0.0001$ (Student's *t*-test). NS, not significant. *n*, sample number. Bar graphs represent mean ± S.E.M. Scale bars: 10 μm (a), 100 μm (c).

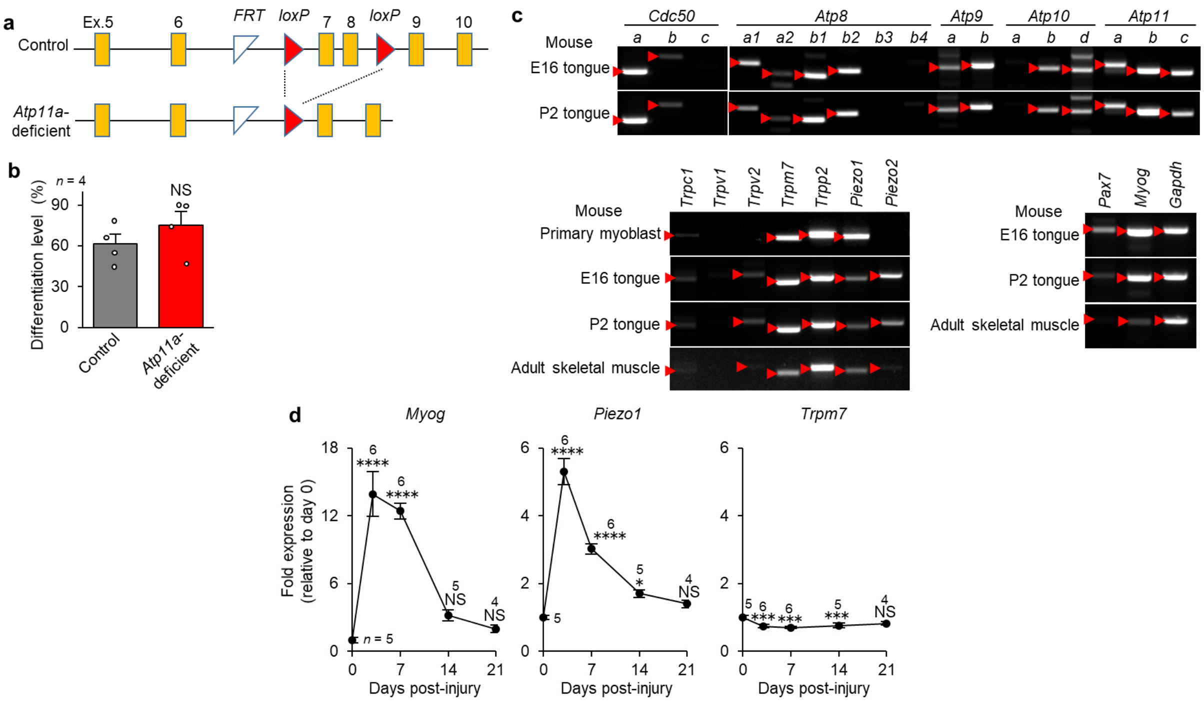

Supplementary Figure 7. Supporting data for a role of ATP11A in morphogenesis during myofibre regeneration. (**a**) The strategy for generation of *Atp11a*-deficient mice. *Atp11a*^{tm1c} was crossed with the *Myf5*-cre transgenic mice, resulting in the removal of exons 7 and 8 in myoblasts. (**b**) Quantification of differentiation in Fig. 8a. (**c**) Semi-quantitative RT-PCR analysis of CDC50 family members, P4-ATPases, mechanosensitive cation channels and differentiation markers in mouse primary myoblasts, tongue (E16 and P2) and adult skeletal muscle. Red arrowheads denote specific bands. **(d)** *In silico* analysis of *Myog* (a myogenic marker), *Piezo1* and *Trpm7* expression during myofibre regeneration after muscle injury. *$P < 0.05$, ***$P < 0.001$ and ****$P < 0.0001$ (Student's *t*-test). NS, not significant. *n*, sample number. Bar graphs represent mean ± S.E.M.

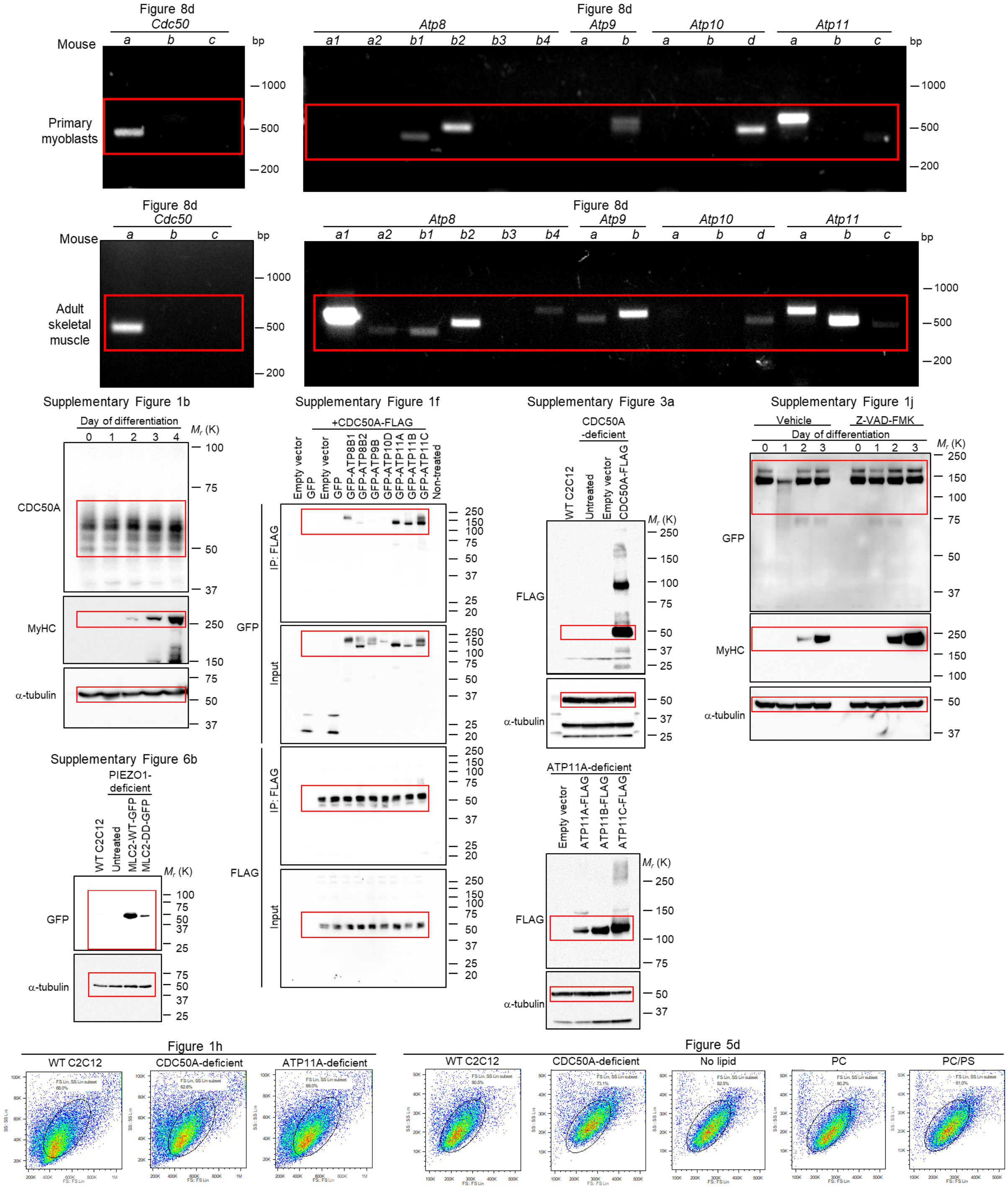

Supplementary Figure 8. Uncropped images of the scans and flow cytometry gating strategies.

Supplementary Table 1. siRNA information.

Target	Species	siRNA ID	Supplier
Non-targeting control	human, mouse	MISSION siRNA universal Negative Control	Sigma-Aldrich
Piezo1 (Fam38a)	mouse	SASI_Mm01_00281158	Sigma-Aldrich
Piezo2 (Fam38b)	mouse	SASI_Mm02_00405285	Sigma-Aldrich
Trpp2 (Pkd2)	mouse	SASI_Mm01_00024215	Sigma-Aldrich
Trpm7	mouse	s81668	Ambion
PIEZO1 (FAM38A)	human	SASI_Hs01_00208584	Sigma-Aldrich
CDC50A (TMEM30A)	human	SASI_Hs01_00054522	Sigma-Aldrich
ATP11A	human	SASI_Hs01_00106358	Sigma-Aldrich

Supplementary Table 2. Antibody information.

Antibodies	Host	Clonality	Usage	Dilution	Catalog number	Supplier
Anti-FLAG	Mouse	Monoclonal, M2	WB	1:1000	F1804	Sigma-Aldrich
			IF	1:500		
Anti-GFP	Rabbit	Polyclonal	WB	1:1000	598	MBL
			IF	1:1000		
Anti-CDC50A	Rabbit	Polyclonal	WB	1:100		In house
Anti-NMIIA	Rabbit	Polyclonal	IF	1:50	M8064	Sigma-Aldrich
Anti-myosin-heavy-chain	Mouse	Monoclonal, MF20	IF	1:500	14-6503-82	eBioscience
Anti-phospho(S19)-MLC2	Rabbit	Polyclonal	IF	1:50	3671	CST
Anti-ROCK1	Rabbit	Polyclonal	IF	1:100	GTX113266	GeneTex
Anti-α-tubulin	Rabbit	Polyclonal	WB	1:1000	PM054	MBL
Anti-PIEZO1	Rabbit	Polyclonal	IF	1:200	NBP1-78446	Novus Biologicals
Anti-PAX7	Mouse	Monoclonal	IF	1:3	528428	DSHB
Anti-laminin	Rabbit	Polyclonal	IF	1:500	L9393	Sigma-Aldrich
HRP-linked-anti-mouse IgG	Sheep	Polyclonal	WB	1:2000-1:4000	NA931V	GE Healthcare
HRP-linked-anti-rabbit IgG	Sheep	Polyclonal	WB	1:2000-1:4000	NA934V	GE Healthcare
Anti-mouse IgG, Alexa Fluor 555	Goat	Polyclonal	IF	1:500	A-21424	Thermo
Anti-rabbit IgG, Alexa Fluor 488	Goat	Polyclonal	IF	1:500	A-11034	Thermo
Anti-goat IgG, Alexa Fluor 488	Donkey	Polyclonal	IF	1:500	A-11055	Thermo

Supplementary Table 3. Mouse qPCR primers.

Target		Test	Sequence	bp
Cdc50a	F	semi-qPCR	CAGTCATTTGAGGGCAATGTGT	470
	R		GCTGCAGTACGCATCCAAAC	
Cdc50b	F	semi-qPCR	GGCCCCGTGTACCTCTACTA	684
	R		AAAGCCCATGACGATGCAGA	
Cdc50c	F	semi-qPCR	ATCCGTTCCAAGTGTCCCAC	531
	R		CCCCCGTCACTGTGTAAGTC	
Atp8a1	F	semi-qPCR	TTGTCTACACTGGCCACGAC	551
	R		TGAGCTCTGCCATTCATCGG	
Atp8a2	F	semi-qPCR	TCATCGAGCTATGGTTCGCC	400
	R		GTCCAAGCTGTCGTCTCCAA	
Atp8b1	F	semi-qPCR	CTGGATCAGGACGTGAGTGAC	391
	R		TGAACTGAAATGCGGACGGA	
Atp8b2	F	semi-qPCR	CGGGCTAACGACCGTGAATA	471
	R		GTTGGTCTCTCCATCCAGCTC	
Atp8b3	F	semi-qPCR	ACGATGAACATGGGACGCTT	386
	R		CTCTGGGTTTCGGACCAACA	
Atp8b4	F	semi-qPCR	GCAGACACGAGGAGTGAACA	612
	R		GGTTTGTTTCCCCGTCAAGC	
Atp9a	F	semi-qPCR	GAAGCGGGTGGACAGTAGGC	517
	R		GCAAGAGCCGTTTTTCTCTGAC	
Atp9b	F	semi-qPCR	GTATCCATGACCGGGCTCTG	561
	R		AAGCCTGTTTGCCCTCCTTT	
Atp10a	F	semi-qPCR	CTGACTGTGGTGTCTGTGCG	598
	R		CTTCTTTTCTTCCCTGCTGAAGAC	
Atp10b	F	semi-qPCR	GTCATCTACGCAGGCCATGA	508
	R		ATTCGCTGCCCACAATGGTA	
Atp10d	F	semi-qPCR	GAACAGTTTCACAGGGCTGC	491
	R		CGGAATCGGCTGAGGTCATT	
Atp11a	F	semi-qPCR	TGACCATCAACGGACAGATGTT	480, 565, 584
	R		TGGAGCACACAACTCTCCCA	
Atp11b	F	semi-qPCR	GTCTCTTGCTTCGTGGAGCC	487
	R		TATTCCACCTGCCCAAGCTCT	
Atp11c	F	semi-qPCR	GCGAGCTAGCTGTCCGCTT	450
	R		AAGCCAATCTTCATACCCCTGC	
Trpc1	F	semi-qPCR	CGTGCGACAAGGGTGACTAT	643
	R		AACATTTTGCACTGACGGGC	
Trpv1	F	semi-qPCR	GGGAGGCCACTCTTACCACA	594
	R		CTTTCCCGTCTCTGGGTCTTT	
Trpv2	F	semi-qPCR	TCCCGAAAGTTCACCGAG	598
	R		TGTAGATGCCTGTGTGCTG	
Trpm7	F	semi-qPCR	ATTTGCCCGTGATACCCCAG	496
	R		CAGCTTTCTGCTTGCACCG	
Trpp2	F	semi-qPCR	GTGTGGTCAGGTTATTGGCG	573
	R		TGCTGAAGTCATCGACCTGG	
Piezo1	F	semi-qPCR	ACATTGCATCCTCGCTGTCA	522
	R		CCTTGGGCTGGGGGTATTTC	
Piezo2	F	semi-qPCR	TTGTTTCAAGGGTTCCGCCT	583
	R		AGCAACTATTGGGGTCGGTG	
Pax7	F	semi-qPCR	CTGGAAGTGTCCACCCCTCT	532
	R		CCACATCTGAGCCCTCATCC	
Myog	F	semi-qPCR	CCCAACCCAGGAGATCATTTG	506
	R		AGGTCAGGGCACTCATGTCT	
Gapdh	F	semi-qPCR	TGAAGGGTGGAGCCAAAAGG	545
	R		GGAAGAGTGGGAGTTGCTGTTG	
Atp8b1	F	qPCR	TGCCGTGTGCTTACTACCTG	208
	R		GGAGATGAGGTCTGCGTAGC	
Atp8b2	F	qPCR	CAGGCCAGTTGAACCTCCTA	198
	R		GCACACTGACCACAATGACC	
Atp9b	F	qPCR	CGAGTTTGTCCATGTTGTGG	193
	R		GAAAGCGCCAAAAGACACTC	
Atp10d	F	qPCR	CCGTGTTTCCATCTCCAGTT	171
	R		CAGAAGACCCAGGTGTTGGT	
Atp11a	F	qPCR	CTGGCGGGTGTTCATTTACT	167
	R		TGACAGTGAGCACCATCACA	
Atp11b	F	qPCR	TTTTGGGCTCCCAGAATATG	246
	R		TTCTTCCAACAGACGCACAC	
Atp11c	F	qPCR	TTACAGTTGGGGCCCTTCTT	193
	R		TATCCAAGGCGAGCTTCAGA	
Piezo1	F	qPCR	ATCCTGCTGTATGGGCTGAC	127
	R		AAGGGTAGCGTGTGTGTTCC	
Piezo2	F	qPCR	CGCTCAGAAATGGTGTGCTA	88
	R		AGATCAAGATGGGCAACAGG	
Trpc1	F	qPCR	AGCCTCTTGACAAACGAGGA	171
	R		TCTTACAGGTGGGCTTACGG	
Trpm7	F	qPCR	AGGATGTCAGATTTGTCAGCAAC	128
	R		CCTGGTTAAAGTGTTCACCCAA	
Trpp2	F	qPCR	AGGTGTTAGGACGGCTGCT	72
	R		CCCTGTGGATCTCACTGTCC	
Ap3d1	F	qPCR	AGGCTCAGAAAAAGGTCCCA	214
	R		AAGGGGTTGTTGGCTTGTTC	
Gapdh	F	qPCR	AAGGTCATCCCAGAGCTGAA	138
	R		CTGCTTCACCACCTTCTTGA	
18S rRNA	F	qPCR	CGCCGCTAGAGGTGAAATTCT	101
	R		CGAACCTCCGACTTTCGTTCT	

Supplementary Table 4. Human qPCR primers.

Target		Test	Sequence	bp
CDC50A	F	semi-qPCR	GAAAAAGAAAGGTATTGCTTGGTG	483
	R		GTAATGTCAGCTGTATTACTACTG	
CDC50B	F	semi-qPCR	CCGACTACCACGTCAAGTTCC	402
	R		AAAGCCGGTGAGGATGCAGAG	
CDC50C	F	semi-qPCR	GGACAGATAAGTATGTCAAATTTC	483
	R		TTTTTTGAAAGTGGGAAAGGCAG	
ATP8A1	F	semi-qPCR	GGCCTGCAGGCAGCTAATTCC	347
	R		GTGTTGAAGTCCAGGGCATTC	
ATP8A2	F	semi-qPCR	GTCACTGCATCAACGCCTTGG	481
	R		TTGCTATCCCGCAGCACCGCTTT	
ATP8B1	F	semi-qPCR	GTGGCCTCCACCAACCGGG	298
	R		CACCTCTATTCCTCTGGTTTTCC	
ATP8B2	F	semi-qPCR	GGGAGAGAGGCCTGAACCTG	331
	R		GAAGTCCAGGATGGCCAGCAG	
ATP8B3	F	semi-qPCR	GCCTGCTGTCCATCACCATGG	354
	R		GTACATGAGGCAAGGGCTCC	
ATP8B4	F	semi-qPCR	GGAAGGCCTTCGGACCTTGG	310
	R		GTCAGTCAGCATGTTGCAGGC	
ATP9A	F	semi-qPCR	GAGGCTCACCTCGAGCTGAAC	271
	R		CCACGCCGCAGTCAGATTCC	
ATP9B	F	semi-qPCR	CAACAGCTGCCGGCTCTGG	373
	R		GATTGCGGTACCATGGACCC	
ATP10A	F	semi-qPCR	CCTTATCCCCAGTCACAGCTG	348
	R		CCGAGTCTGCCTCTTGGTACC	
ATP10B	F	semi-qPCR	CAGGATCCAGCAACTATGAGAAG	318
	R		GGACACCATGACAGAGTTGCAG	
ATP10D	F	semi-qPCR	CCGAGCCACACCGCTGCAG	351
	R		CAGTAATCAGTCATGGATGTTCC	
ATP11A	F	semi-qPCR	GCTGCTGCAGGCTGCCAAAG	355
	R		GTCTCTGGTCAGGCTCCCGC	
ATP11B	F	semi-qPCR	CCTGTCAAGTGGTTCTGCTTGG	339
	R		CTTTAACAAGTAGATGAGTCCATTG	
ATP11C	F	semi-qPCR	GGAACGTAATGCAATGGATGGG	349
	R		GGTTAGTTCTAAGAGCTCAGTG	
TRPC1	F	semi-qPCR	CAGTGGGAACGACTCATCCT	451
	R		TCTGCAGACTGACAACCGTAG	
TRPV1	F	semi-qPCR	ATGACAGTGTGATGGAGAGTC	422
	R		AACAGGGCTGACTGTGATGG	
TRPV2	F	semi-qPCR	TCTTCCTTTTCGGCTTCGCT	575
	R		CTCGAGAGTTCGAGGGACAC	
TRPM7	F	semi-qPCR	CCTGTTCATTCAAAACAAGCAGAAA	407, 410
	R		GCTCTTCGTAAACCTCCTCCC	
TRPP2	F	semi-qPCR	CGGCTGATGGCTGGCTG	530
	R		CTTGTTCCCCAGAGACCTCG	
PIEZO1	F	semi-qPCR	CACCAACCTCATCAGCGACT	414
	R		AGCGACAGCATGTTCTTGGA	
PIEZO2	F	semi-qPCR	TCTGCGGAGGCATGTTCTTC	432
	R		GGGCCAGTCTGTAGATGGTGT	
PIEZO1	F	qPCR	CAATGAGGAGGCCGACTACC	106
	R		GCACTCCTGCAGTTCGATGA	
CDC50A	F	qPCR	CGATGGCGATGAACTATAACGC	252
	R		CGGTATAATCAATCTCGATCTC	
ATP11A	F	qPCR	CACAGAGATACCCAGACAACAGG	131
	R		CAACTGCACCAGAAATATGATAAGG	
GAPDH	F	qPCR	ATGGGGAAGGTGAAGGTCG	108
	R		GGGGTCATTCATGGCAACAATA	
