# Supplementary material for: Cell surface flip-flop of phosphatidylserine is critical for PIEZO1-mediated myotube formation
Source: Nat Commun. 2018 May 24;9:2049. doi: 10.1038/s41467-018-04436-w (PMC5967302; doi:10.1038/s41467-018-04436-w)
Supplement: Supplementary file 3 — Description of Additional Supplementary Information [file 41467_2018_4436_MOESM3_ESM.docx]

**Description of Additional Supplementary Files**

File Name: Supplementary Movie 1

Description: **Time-lapse imaging of myotube formation in WT C2C12 cells.** Differential interference contrast (DIC) images were taken at 1 frame per 20 min from 2 days after induction of differentiation and played at 5 frames/s. Acquisition time, h:min. Scale bar, 50 μm. Selected frames are shown in Supplementary Fig. 1g.

File Name: Supplementary Movie 2

Description: **Time-lapse imaging of myotube formation in CDC50A-deficient C2C12 cells.** DIC images were taken at 1 frame per 20 min from day 2 after induction of differentiation and played at 5 frames/s. Acquisition time, h:min. Scale bar, 50 μm. Selected frames are shown in Supplementary Fig. 1g.

File Name: Supplementary Movie 3

Description: **Time-lapse imaging of myotube formation in ATP11A-deficient C2C12 cells.** DIC images were taken at 1 frame per 20 min from day 2 after induction of differentiation and played at 5 frames/s. Acquisition time, h:min. Scale bar, 50 μm. Selected frames are shown in Supplementary Fig. 1g.

File Name: Supplementary Movie 4

Description**: Time-lapse imaging of myotube formation in PIEZO1-deficient C2C12 cells.** DIC images were taken at 1 frame per 20 min from day 2 after induction of differentiation and played at 5 frames/s. Acquisition time, h:min. Scale bar, 50 μm.
