# Supplementary material for: Cell surface flip-flop of phosphatidylserine is critical for PIEZO1-mediated myotube formation
Source: Nat Commun. 2018 May 24;9:2049. doi: 10.1038/s41467-018-04436-w (PMC5967302; doi:10.1038/s41467-018-04436-w)
Supplement: Supplementary file 2 — Peer Review File [file 41467_2018_4436_MOESM2_ESM.pdf]

Editorial Note: This manuscript has been previously reviewed at another journal that is not operating a transparent peer review scheme. This document only contains reviewer comments and rebuttal letters for versions considered at Nature Communications. Mentions of prior referee reports have been redacted.

Reviewers' comments:

Reviewer #1 (Remarks to the Author):

The authors have been mostly responsive after initial reviewer comments, and added new data to the manuscript. I am convinced that CDC50A, ATP11a, and PIEZO1 play a role in muscle formation in vitro, which is novel. However, there are some inconsistencies about mechanistic details and overall in vivo relevance.

1. Whether this pathway is important in vivo is still not clear. Their interpretation is based solely on injuring adult Atp11a-deficient muscle and analyzing longitudinal sections to visualize 'fused fibers'. I am not certain if the areas notated by the red arrows in Fig. 5j truly represent fused fibers, or they indicate the beginning of a new fiber or fiber branching. I realize the difficulty of this analysis especially after injury - one possibility is the isolation of single fibers. Additionally, it would be helpful to understand the general histology of these muscles.
2. It is still odd that the authors strongly interpret in vivo relevance from Fig. 5j but gloss over the fact there is no developmental phenotype.
3. Mechanistically, it is unclear if this pathway is operating in myoblasts or myotubes, or both. The potential main in vivo phenotype of 'fused fibers' indicates that the pathway has an effect on myofibers and not myoblasts. However, throughout the manuscript the authors suggest that the proteins are working in myoblasts or 'myogenic cells'. It is further confusing that PIEZO1 is presented as being expressed on satellite cells and not the myofiber (Fig. 5c-e). For me, the data indicate that the pathway is likely functional on the growing myofiber to prevent dysregulated fusion/elongation. Overall, what is missing from the plethora of data is a coherent picture of where these proteins are normally functioning to control fusion/myotube elongation.
4. The inclusion of so much data, and the divergent claims associated with the data, makes the manuscript difficult to read. There are panels of figures that are not explained in the text including Fig. 1g and 5e. I assume there is little explanation due to space constraints, but it still makes for a less than ideal manuscript. I think a significantly altered manuscript with less overall data would result in a more streamlined message.
5. The authors write that a reduction of this pathway leads to no significant accumulation of cortical F-actin (Fig. 1i and 2e) and loss of NMIIA. No question that NMIIA is reduced and this is quantified nicely, but I don't understand why they interpret a reduction in cortical F-actin. There is clearly phalloidin staining in the experimental cells and while it may be structurally disrupted, it would require some quantification for interpretation.

Reviewer #2 (Remarks to the Author):

The authors responded to most queries, and performed a substantial number of experiments. Overall, the manuscript improved significantly.

In vivo relevance was a significant problem, raised by more than one reviewer. The demonstration of aberrant fusion of myofibres form Atp11a knockout mice in regenerating muscle addresses this to some extent.

I still believe that the in vivo relevance of Piezo1 in myoblast fusion is not proven by the manuscript, given the lack of documented muscle phenotype either in Piezo1 knockout mice, or in

Piezo1 gain or loss of function mutations in humans. This needs to be addressed at one point in the future, by generating muscle specific Piezo1 knockout mice, but it is probably beyond the scope of the manuscript.

Reviewer #3 (Remarks to the Author):

The authors have done a good job addressing my concerns and I now favor publication of this important study.

Point-by-point response to the reviewers' comments:

Reviewer #1 (Remarks to the Author):

The authors have been mostly responsive after initial reviewer comments, and added new data to the manuscript. I am convinced that CDC50A, ATP11a, and PIEZO1 play a role in muscle formation in vitro, which is novel. However, there are some inconsistencies about mechanistic details and overall in vivo relevance.

We thank the reviewer for the careful reading of our manuscript and the constructive comments. As suggested by the reviewer, we performed a series of experiments, and the changes made in the revised manuscript are as follows:

1. Whether this pathway is important in vivo is still not clear. Their interpretation is based solely on injuring adult Atp11a-deficient muscle and analyzing longitudinal sections to visualize 'fused fibers'. I am not certain if the areas notated by the red arrows in Fig. 5j truly represent fused fibers, or they indicate the beginning of a new fiber or fiber branching. I realize the difficulty of this analysis especially after injury - one possibility is the isolation of single fibers. Additionally, it would be helpful to understand the general histology of these muscles.

1) In this study, we evaluated the morphology of regenerating myofibres in *Atp11a*-deficient tibialis anterior muscle at day 14 after the injury, at which period early regeneration processes such as myoblast fusion and nascent myofiber formation have almost completed. Our immunohistological analyses demonstrated that the 'fused fibers' are evident in the longitudinal sections of the regenerating *Atp11a*-deficient muscles (Fig. 8i, j in the revised manuscript). As observed in the present study, these fused fibers (branched fibers) are frequently observed in the pathogenic conditions: regenerating muscles in mdx mouse, a disease model for muscular dystrophy (Pichavant C and Pavlath GK, *Skeletal muscle*, 2014), and in mutant mice that display the *in vitro* hyperfusion phenotype (Charrin S et al., *Nat Commun* 2013). We have also examined the cross sections of the regenerating myofibres and have observed the significant increase in the number of myofibres positive for embryonic myosin in the *Atp11a*-deficient regenerating muscle (Appended figure). Embryonic myosin is a hallmark of regenerative myogenesis, which is detected in fusion-competent cells as well as newly formed regenerating myofibres at 2-3 days after injury and persist for 2-3 weeks (Hindi SM et al., *Sci. Signal.* 2013, Schiaffino S et

al., *Skeletal Muscle*, 2015). Embryonic myosin is suggested to play a structural role in myofibril formation during muscle regeneration, but its physiological function is still remained to be clarified. Although these observations may imply that the embryonic myosin-positive myofibres are involved in the formation of the 'fused fibers', we have not included the results in the revised manuscript because further detailed analyses are required to clarify the physiological function of the embryonic myosin-positive myofibres.

Appended Figure

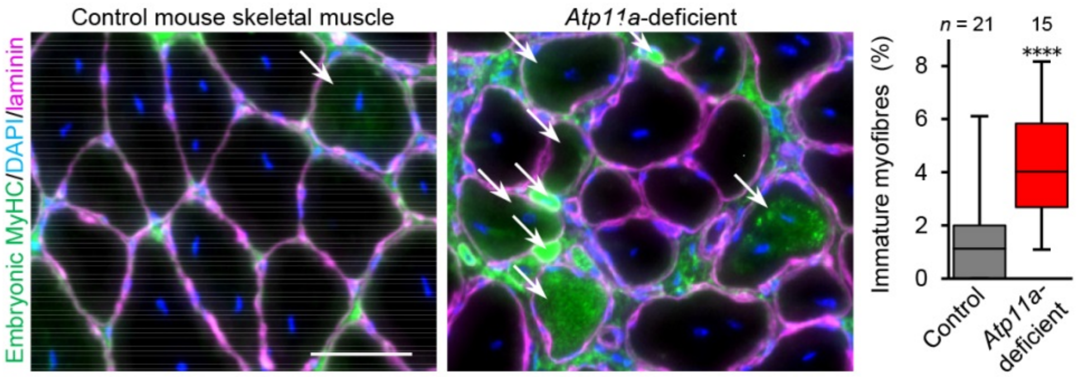

Increased number of embryonic myosin-positive myofibres in *Atp11a*-deficient regenerated muscle after cardiotoxin-induced degeneration.

Cross-sections prepared from control (left) and *Atp11a*-deficient adult muscle (right) were probed with anti-laminin antibody (basal lamina, magenta), anti-embryonic MyHC antibody (immature myofibre, green), and DAPI (nuclei, blue). Arrows indicate immature regenerating myofibres expressing embryonic MyHC. Scale bar: 50 μm.

We have added the following sentences in the 'Result' section of the revised manuscript:

[P14, L323]

‘In this study, we evaluated the morphology of regenerating myofibres in *Atp11a*-deficient tibialis anterior (TA) muscle. Cardiotoxin, a myotoxic agent that causes degeneration and concomitant regeneration of myofibres[46], was injected into TA muscle of *Atp11a*-deficient mice. The regenerating muscle tissues were harvested at 2 weeks post-cardiotoxin injection, then morphological analysis was conducted by staining longitudinal sections from the cardiotoxin-injected muscles with anti-laminin antibody (for the extracellular matrix), anti-MyHC antibody (for myofibres) and DAPI (for nuclei). Our immunohistological analyses demonstrated that, upon cardiotoxin administration, abnormal myofibres that fused with each other were evident in cardiotoxin-injected *Atp11a*-deficient TA muscle (Fig. 8i, j), as observed in

regenerating muscles of mutant mice that display the *in vitro* hyperfusion phenotype[47]. These results suggest that ATP11A-mediated PIEZO1 activation plays a crucial role in proper morphogenesis during myofibre regeneration.'

2) Due to the fragility of damage myofibres, the isolation of single fibers from the regenerating muscle is extremely difficult and we have not yet succeeded in the isolation.

3) As suggested by the reviewer, we have included the sentences that explain the general histology of muscle regeneration in the revised manuscript:

[P14, L319]

'Adult skeletal muscle has the ability to efficiently regenerate after different types of injury. Muscle regeneration is mediated by satellite cells residing beneath the basal lamina of muscle fibres, which are activated after injury and undergo myogenic commitment to become fusion-competent myoblasts[44]. The resulting myoblasts fuse with each other to generate nascent syncytia that mature into functional myofibres[10].'

2. It is still odd that the authors strongly interpret in vivo relevance from Fig. 5j but gloss over the fact there is no developmental phenotype.

Since systemic knockout of *Atp11a* resulted in lethality during embryogenesis, we generated myoblast-specific conditional *Atp11a*-deficient mice and examined the muscle phenotypes. Unfortunately, no obvious morphological or behavioural abnormalities were observed between the control and *Atp11a*-deficient mice. To further evaluate the physiological function of the ATP11A-PIEZO1 axis in myogenesis, we performed detailed analyses on the stage- and tissue-specific expression of ATP11A and PIEZO1 in mouse muscle tissues. As shown in Supplementary Fig. 7c in the revised manuscript, we found that a variety of cell surface P4-ATPases including *Atp8a1*, *Atp8a2*, *Atp8b1*, *Atp8b2*, *Atp9a*, *Atp10d, Atp11a* and *Atp11c* were expressed in developing muscle. It is likely that these P4-ATPases may functionally compensate for the defective expression of ATP11A during developmental myogenesis.
Furthermore, no significant expression of PIEZO1 was detected in developing and adult muscle, respectively (Fig. 8e and Supplementary Fig.7c). We, however, found robust expression of PIEZO1 in primary myoblasts as well as in Pax7-positive satellite cells, a population of myogenic progenitor cells in adult muscle (Fig. 8e-h). These observations

prompted us to examine the physiological function of the ATP11A-PIEZO1 axis in muscle regeneration using a cardiotoxin-injection model (Millay DP et al., *Nature* 2013; Charrin S et al., *Nat Commun* 2013; Shi X et al., *Genes Dev* 2006).

We have added the sentences [from **P13 L288** to **P14 L318**] in the 'Result' section of the revised manuscript.

3. Mechanistically, it is unclear if this pathway is operating in myoblasts or myotubes, or both. The potential main in vivo phenotype of 'fused fibers' indicates that the pathway has an effect on myofibers and not myoblasts. However, throughout the manuscript the authors suggest that the proteins are working in myoblasts or 'myogenic cells'. It is further confusing that PIEZO1 is presented as being expressed on satellite cells and not the myofiber (Fig. 5c-e). For me, the data indicate that the pathway is likely functional on the growing myofiber to prevent dysregulated fusion/elongation. Overall, what is missing from the plethora of data is a coherent picture of where these proteins are normally functioning to control fusion/myotube elongation.

First of all, we would like to apologize for any confusion caused by the ambiguous terminology of 'myogenic cells', which were used to represent the group of cells that participate in the whole myogenic processes including satellite cells, myoblasts, myotubes, and even the growing myofibers as the reviewer pointed out. We have deleted the term "myogenic cells" in the revised manuscript.

1) Based on the present observations, we conclude that the ATP11A-PIEZO1 axis is operating in both myoblasts and myotubes. During the course of muscle regeneration, satellite cells are activated after injury and undergo myogenic commitment to become fusion-competent myoblasts. The resulting myoblasts fuse with each other to generate nascent multinucleated cells (i.e. myotubes) that are maturated into functional myofibres. As demonstrated in the Supplementary Movies 2-4 of PS flippase- and PIEZO1-deficient C2C12 cells, both myoblasts and myotubes undergo uncontrolled fusion with adjacent myotubes. We demonstrated that during myotube formation, the ATP11A-PIEZO1 axis plays a crucial role in the assembly of the cortical actomyosin fibres, which prevents uncontrolled fusion of adjacent myotubes and generates a lateral compression force to support polarized elongation (Mukai and Hashimoto, *Exp. Cell Res*. 2008; Abmayr and Pavlath, *Development*

2012). We also showed that PIEZO1 remains highly expressed during nascent myotube formation *in vitro* (Supplementary Fig. 4a) and that PIEZO1 expression is up-regulated during regenerative myofiber formation (Supplementary Fig. 7d). These results suggest that the incomplete actomyosin formation may cause the sporadic fusion of myofibres in the *Atp11a*-deficient muscle, resulting in the formation of the abnormally fused myofibres.

We have added the following sentences in the 'Result' section of the revised manuscript:
[P8 L158]
'During myotube formation, assemblies of F-actin and non-muscle myosin IIA (NMIIA) create actomyosin fibres underneath the plasma membrane, which prevents uncontrolled fusion of adjacent myotubes and generates a lateral compression force to support polarized elongation[10, 32].'

[P8 L174]
'Of the Ca^{2+} channels examined, siRNA-mediated depletion of PIEZO1[24, 25], a mechanosensitive Ca^{2+} channel predominantly expressed during myotube formation, resulted in the formation of sheet-like syncytia (Supplementary Fig. 4a, b), showing a morphological phenotype quite similar to that observed in the ATP11A- and CDC50A-deficient cells (Fig. 1a).'

[P13 L282]
'These results collectively indicate that flippase-mediated PS translocation at the plasma membrane regulates PIEZO1 activation, which promotes RhoA/ROCK-mediated phosphorylation of MLC2 and subsequent assembly of cortical actomyosin fibres, thereby controlling fusion and polarized elongation during myotube formation.'

2) To unravel the *in vivo* function of the ATP11A-PIEZO1 axis in muscle regeneration, further experimentations including generation of muscle type-specific knockout mice of PIEZO1 and transgenic mice expressing Ca^{2+} indicator to trace the PIEZO1 function, and *in vivo* visualization and manipulation of PS are required. Since these analyses will require at least several years of intensive effort, we consider these experimentations to be far beyond the scope of a single report, as reviewer #2 mentioned.

4. The inclusion of so much data, and the divergent claims associated with the data, makes the manuscript difficult to read. There are panels of figures that are not explained in the text including Fig. 1g and 5e. I assume there is little explanation due to space constraints, but it still makes for a less than ideal manuscript. I think a significantly altered manuscript with less overall data would result in a more streamlined message.

1) As suggested by the reviewer, we have reorganized figures and have added schematic models in figures (Fig. 3f; Fig. 5a, 5c; Fig. 6b, 6d, 6f; Fig. 7a in the revised manuscript). We have also added 'Introduction' and 'Discussion' sections and have thoroughly revised the manuscript as highlighted in the manuscript.

2) We have deleted supplementary fig. 3f, 3g, 3i, and 3p in the original manuscript, because the descriptions related to the structural insights into the PS-PIEZO1 interaction have been toned down in the revised manuscript.

5. The authors write that a reduction of this pathway leads to no significant accumulation of cortical F-actin (Fig. 1i and 2e) and loss of NMIIA. No question that NMIIA is reduced and this is quantified nicely, but I don't understand why they interpret a reduction in cortical F-actin. There is clearly phalloidin staining in the experimental cells and while it may be structurally disrupted, it would require some quantification for interpretation.

As suggested by the reviewer, we have included the quantification data on the cortical assembly of F-actin in the revised manuscript, as shown in Fig. 1j and Fig. 2f.

We have added the following sentences in ‘Results’ section of the revised manuscript:
[P8 L161]
‘Both F-actin and NMIIA were enriched at the lateral cortex of wild-type (WT) C2C12 myotubes, but neither significant accumulation of F-actin nor NMIIA to the cell periphery was observed in the sheet-like syncytium of the CDC50A- and ATP11A-deficient cells (Fig. 1i). Analysis of actomyosin localization by quantifying cortex/cytoplasm ratios[33] clearly demonstrated the suppressed assembly of cortical F-actin and NMIIA in the sheet-like syncytium, which accumulated in unfused mononuclear cells (Fig. 1i, j).’

Reviewer #2 (Remarks to the Author):

The authors responded to most queries, and performed a substantial number of experiments. Overall, the manuscript improved significantly.

In vivo relevance was a significant problem, raised by more than one reviewer. The demonstration of aberrant fusion of myofibres form Atp11a knockout mice in regenerating muscle addresses this to some extent.

I still believe that the in vivo relevance of Piezo1 in myoblast fusion is not proven by the manuscript, given the lack of documented muscle phenotype either in Piezo1 knockout mice, or in Piezo1 gain or loss of function mutations in humans. This needs to be addressed at one point in the future, by generating muscle specific Piezo1 knockout mice, but it is probably beyond the scope of the manuscript.

We thank the reviewer for careful consideration of our manuscript. We strongly agree with the suggestion that the phenotypic evaluation on muscle-specific *Piezo1*-deficient mice would be urgently required. We will conduct a series of experiments on *Piezo1*-deficient muscle in the near future, to completely prove the *in vivo* relevance of our findings.

Reviewer #3 (Remarks to the Author):

The authors have done a good job addressing my concerns and I now favor publication of this important study.

We thank the reviewer for careful consideration of our manuscript and are happy to see that she/he shares our excitement about the importance of our findings.

REVIEWERS' COMMENTS:

Reviewer #1 (Remarks to the Author):

The authors have been as responsive as possible for this manuscript. Many of the criticisms will have to be dealt with by the authors with future experiments.
